# Coordinated hippocampal–entorhinal representations support human context-dependent spatial navigation

**DOI:** 10.1371/journal.pbio.3003398

**Published:** 2025-09-17

**Authors:** Pengcheng Lv, Dong Chen, Chao Zhang, Wei Duan, Pingping Lu, Kai Zhang, Liang Wang

**Affiliations:** 1 State Key Laboratory of Cognitive Science and Mental Health, Institute of Psychology, Chinese Academy of Sciences, Beijing, China; 2 Department of Psychology, University of Chinese Academy of Sciences, Beijing, China; 3 Department of Neurosurgery, Beijing Tiantan Hospital, Capital Medical University, Beijing, China; University of Glasgow, UNITED KINGDOM OF GREAT BRITAIN AND NORTHERN IRELAND

## Abstract

To execute goal-directed behavior, retrieval of both context and object information is crucial. Despite the ubiquity of such contextual computations in daily navigation, the neural mechanisms underlying this process in humans and its connection to behavior remain largely elusive. Leveraging intracranial electroencephalography (iEEG) recorded from epilepsy patients (*N* = 31) engaged in a context-dependent spatial navigation task, we uncovered distinct oscillatory patterns in the hippocampus (HC) and entorhinal cortex (EC) that represented context and object information, respectively. Notably, the covariation of these neural representations predicted behavioral performance. Furthermore, both representations were primarily driven by low-frequency oscillations (2–8 Hz). The synchronization of low-frequency oscillations between HC and EC was associated with enhanced object representations in the EC. These findings highlight the importance of low-frequency neural dynamics in mediating both local representations and interregional interactions within the hippocampal–entorhinal circuit during context-dependent spatial navigation.

## Abstract 2

During task performance, the brain must integrate information about goal-related objects with contextual details. This study shows that participants' performance on a spatial navigation task is related to the synchronization of activity between the hippocampus and the entorhinal cortex, each encoding different types of task-relevant information.

## Introduction

Individuals encounter a variety of spatial maps in daily life. To perform goal-oriented behaviors, it is essential to accurately retrieve the corresponding context, location, object information, and other details based on the experiences associated with memory formation. For example, when recalling the location of an object, such as a computer, individuals typically retrieve contextual details, such as “It was on the desk in the office, near the window,” or “It was in the bedroom at home, north of the bed,” illustrating context-dependent spatial recall.

This ability has been proposed to be mediated by the distinctive neural activity in the hippocampus (HC) and entorhinal cortex (EC) [1]. One of the most significant discoveries involves hippocampal place cell remapping across spatial contexts in rodents [2–4]. Recently, human neuroimaging studies using virtual navigation tasks have revealed distinct spatial map patterns in hippocampal structures through multivariate analysis methods, such as classifiers and representational similarity analysis (RSA) [5,6]. These findings suggest that the hippocampus encodes contextual information. As for object information, both human neuroimaging study [7] and single-cell recording [8] in rodents support its encoding in the entorhinal cortex.

During memory retrieval, it is hypothesized that hippocampal ensembles reactivate corresponding contextual information upon receiving relevant cues [9,10]. This reactivation is thought to coordinate with memory item representations within the EC [11,12]. Nevertheless, how this process operates in humans at the neural level and how it relates to behavior remains unresolved. Specifically, the precise coupling of neural dynamics between the HC and EC during context-dependent spatial memory retrieval remains unclear, particularly at subsecond timescale. Emerging evidence suggests that neural oscillations may play a crucial role in this process. For example, a rodent study has demonstrated that distinct gamma oscillations in subregions of the EC carry signals for objects and locations, synchronizing with theta rhythms in the HC [13]. This phase-locked coordination may constitute a fundamental mechanism for integrating contextual and object information during memory retrieval.

Intracranial electroencephalography (iEEG) recordings from epilepsy patients provide a unique opportunity to investigate these issues, owing to high spatial and temporal resolution of iEEG. This method has been successfully employed to track the dynamic representations of specific content at subsecond timescales and to explore how these representations are organized to facilitate inter-regional information transfer [14,15]. To address these questions, we recruited epilepsy patients (*N* = 31) to complete a context-dependent spatial navigation task and analyzed the electrophysiological activity in the HC and EC. Specifically, we measured the oscillatory reactivation patterns of contextual and object information, examined the coherence of these reactivation patterns, as well as their relationship with behavioral performance. Our results reveal that: (1) during translational movement, navigation contexts and object information are detected at the mesoscopic local field potential (LFP) level within the HC and EC, respectively; (2) these neural patterns covary with successful navigation, enabling the prediction of behavioral performance; (3) both representations are predominantly driven by low-frequency oscillations; and (4) the synchronization of low-frequency oscillations between the HC and EC predicts the strength of object representations in the EC, but not context representations in the HC.

## Results

### Behavioral results

**Fig 1:**
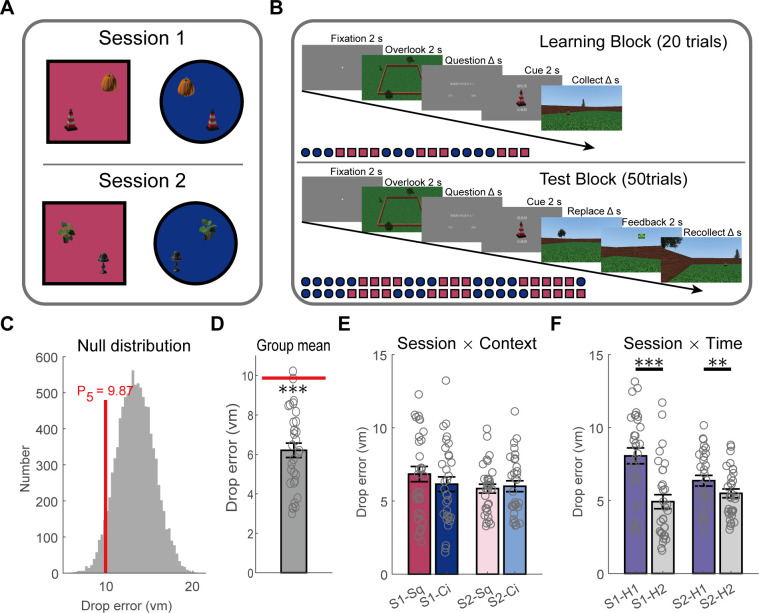
Navigation task and behavioral results. **(A)** Schematic illustration of a virtual navigation task. The experiment consisted of two sessions (Session 1 and Session 2). In each session, participants were required to learn and remember the different locations of two same objects in both the square and circle environments. **(B)** Trial structure. Each session includes 20 learning trials (the learning block) and 50 test trials (the test block). After completing 3–5 consecutive trials in one environment, participants switch to the other environment, ensuring an equal number of trials in the two environments. All participants follow the same sequence, as depicted in the bottom left corner. **(C)** The surrogate distribution of drop error, with a 5th percentile value of 9.87 vm as the chance level. **(D)** The drop error of the participants was significantly lower than the chance level (red line). **(E)** In each session, there was no significant difference in the drop errors between the square and circle environments. **(F)** In both sessions, the drop error in the second half of the trials was significantly less than the drop error in the first half of the trials. *** *p* < 0.001, ** *p* < 0.01. Hollow dots are individual subjects, and error bars indicate standard error of the mean (SEM) across participants in panels **D–F**. The data underlying this figure can be found in https://doi.org/10.5281/zenodo.17017876.

We recruited 31 epilepsy patients to complete a context-dependent spatial navigation task (Fig 1A and 1B). The experiment comprised two sessions, in which participants were required to memorize two identical objects positioned at distinct locations within square and circular environments (see “Experimental task” for details).

To assess task performance, we first established a surrogate distribution of drop errors [16], with the fifth percentile of the distribution (9.87 vm) serving as a critical threshold for determining significant deviation from chance level at the *p* = 0.05 threshold (Fig 1C; see “Behavioral data analysis” for details). As illustrated in Fig 1D, the group mean drop error was significantly lower than the critical threshold (*t*_30 _= −10.004, *p* < 0.001), indicating above-chance performance. Subsequently, we examined potential differences in drop errors between sessions and across environments. A two-way repeated measures ANOVA revealed that neither the main effect of session (*F*_(1,30)_ = 3.67, *p* = 0.067) nor the main effect of environment (*F*_(1,30)_ = 1.09, *p* = 0.305) was significant, nor the interaction effect between them (*F*_(1,30)_ = 2.75, *p* = 0.107; Fig 1E). These results indicated that participants exhibited overall good performance (see S1–S3 Figs for drop positions, return trajectories, and trial durations), with no significant behavioral differences observed between environments despite a marginal trend across sessions.

Next, we assessed whether the participants exhibited a learning effect over the time course of the task. For each session, we divided the trials into two equal halves: the first 25 trials (H1) and the last 25 trials (H2), and calculated the mean drop errors for each half. A repeated measures ANOVA (Fig 1F) showed that the main effect of session was not significant (*F*_(1,30)_ = 3.67, *p* = 0.067), while the main effect of time was significant (*F*_(1,30)_ = 50.73, *p* < 0.001), and their interaction was also significant (*F*_(1,30)_ = 25.59, *p *< 0.001). Simple effects analysis revealed significant performance improvements (reduction in drop errors) from the first to the second half in both session 1 (*t*_30_ = 7.09, *p* < 0.001) and session 2 (*t*_30_ = 3.46, *p* = 0.0016). The decrease in drop error in the second half of the trials compared to the first half was significantly greater in the first session (*t*_30_ = 5.06, *p* < 0.001). These results provide robust evidence for the presence of learning effects across both sessions of the spatial navigation task, with more pronounced improvements occurring during the first session.

Participants achieved near-perfect context identification (only 2.61% incorrect trials across all participants) during Overlook period, with post-experiment debriefing confirming errors stemmed from button-press slips rather than genuine context confusion. Moreover, navigation accuracy (drop error) remained equivalent between correct and incorrect trials (*t*_20_ = −0.644, *p* = 0.527), even after randomly subsampling correct trials to match the number of incorrect trials (*p*_subsample_ = 0.9804, S19 Fig). These results confirm that errors during Overlook main reflect motor execution noise, not fundamental failures in context processing. Thus, we retained incorrect trials in subsequent analyses.

### Context representations in HC

**Fig 2:**
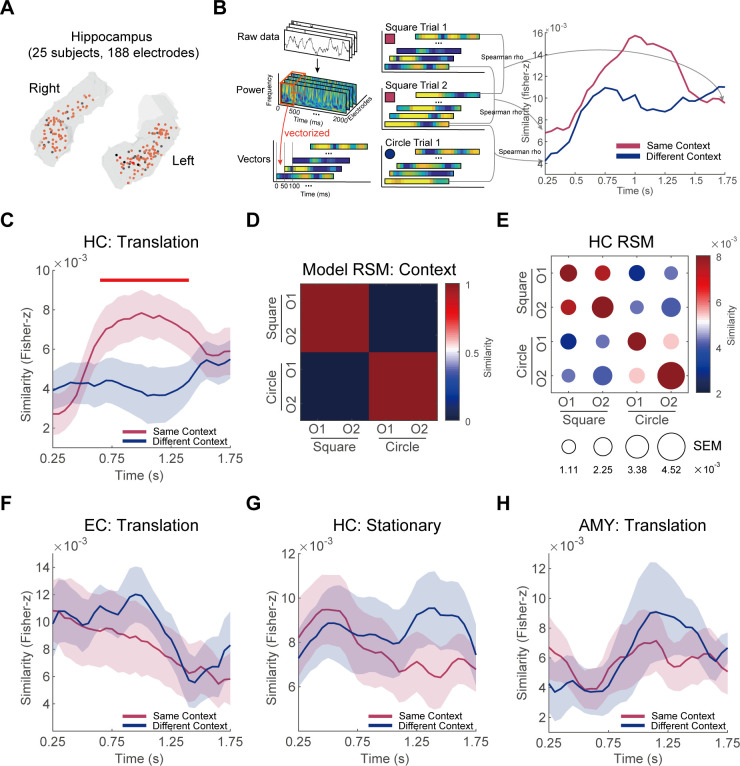
Context representations in HC. **(A)** The positions of HC electrodes projected onto the MNI152 template from all 25 participants. The red dots are task-selective electrodes (*N* = 155). The black dots are non-task-selective electrodes (*N* = 33). **(B)** A schematic illustration of RSA of HC context representations. **(C)** During the period from 0.65 to 1.45 s after the translation onset, the similarity of Same Context (the red line) was significantly greater compared to the similarity of Different Context (the blue line) in HC. The horizontal red line marked the time window where these significant differences were observed. **(D)** The representational similarity matrix (RSM) for context representation model. **(E)** Group-level RSM of HC. Circle color represents the mean representational similarity, while the size denotes SEM across participants. **(F)** During the translation epochs, EC did not exhibit context representation. **(G)** During the stationary epochs, HC did not show context representation. **(H)** During the translation epochs, the amygdala did not exhibit context representation. The shaded areas represent SEM across participants in panels **C, F, G, and H**.

Next, we examined the representations of environmental context in the HC during memory retrieval. Our analysis included data from 25 participants, encompassing a total of 188 hippocampal electrodes (Fig 2A, see details in S1 Table). To more clearly elucidate the effect [17], we focused our primary analysis on good trials, defined as those with drop errors significantly below chance level, across 155 task-selective electrodes (see “Representational similarity analysis” for details).

Using representation similarity analysis (RSA) (Fig 2B) [14,18,19], we identified significantly greater neural pattern similarity of Same Context (red line in Fig 2C) compared to that of Different Context (the blue line in Fig 2C) during the translation epochs in the HC. This context-specific differentiation emerged between 0.65 and 1.45 s following translation onset (*p*_cluster_ = 0.001). To elucidate the context representations in HC more clearly, we categorized trials into four categories based on environmental and object identities (2 environments × 2 objects), reflecting the task structure where participants memorized two identical objects at different locations across two environments. We computed pairwise similarities and averaged them across the significant time window identified in Fig 2C, generating a 4 × 4 representational similarity matrix (RSM) for each participant. Fig 2D illustrates the hypothetical model of context representations, while Fig 2E displays the group-level average of RSM in HC. These findings collectively suggest a pronounced context representation in HC. In contrast, we did not find context representation in EC during the translation epochs (Fig 2F). We also checked hippocampal data from the 10 participants with simultaneous HC and EC electrode coverage, which revealed context representations (albeit weaker effects with uncorrected significance, *p*_cluster_ = 0.11) but no object representations in HC of these participants (S24 Fig).

To validate the specificity of context representation in HC, we conducted additional analyses across different conditions and brain regions. Firstly, we confirmed the absence of context representation in the HC during stationary epochs (Fig 2G). Secondly, we examined the amygdala, a neighboring structure to the HC, and found no evidence of context representation during translation epochs (Fig 2H). To further validate robustness of our RSA results, we conducted a series of control analyses. Firstly, we extended our RSA to include translation epochs from all trials (S4 Fig). Secondly, we expanded our electrode selection to encompass all electrodes within the regions of interest (ROIs), regardless of task selectivity (S5–S6 Figs). Third, we systematically varied the temporal parameters for neural vector construction, modifying the time window from our initial 500 ms width with 50 ms steps to: (a) 200 ms width with 20 ms steps (S7–S8 Figs), and (b) 100 ms width with 10 ms steps (S9–S10 Figs). As a comparison, we analyzed hippocampal activity during the translation epochs of bad trials (drop errors significantly greater than chance level). The results revealed that, in these trials, HC exhibited no significant context or object representations (S13A–S13B Fig). Notably, a direct statistical comparison of HC context representation strength between good and bad trials did not reveal a significant difference (all *p* values > 0.05), a finding likely attributable to near-ceiling performance and a reduced number of bad trials limiting statistical power. These control analyses consistently replicated our primary findings, demonstrating robust context representation in HC while confirming its absence in EC.

To rule out the possibility that HC context effects were solely driven by visual similarity, we applied identical analytical methods to electrodes in temporal and occipital regions. Crucially, if visual similarity between contexts during translation epochs was sufficiently low, we should have observed significantly greater neural pattern similarity for Same Context versus Different Context in occipital electrodes, particularly under visual confound assumptions. However, results demonstrated no detectable context representations in either region (S18 Fig). This provides critical evidence that cross-environment differences in hippocampal activity arise from contextual processing rather than low-level visual differences.

Furthermore, to investigate potential functional specialization along the hippocampal longitudinal axis [20], we divided hippocampal electrodes into anterior and posterior regions using the MNI152 coordinates. Using a *y*-coordinate threshold of −21 mm [20], we classified electrodes with *y* ≥ −21 mm as anterior hippocampus (97 electrodes from 23 participants) and those with *y* < −21 mm as posterior hippocampus (58 electrodes from 23 participants). Our analysis revealed that both anterior and posterior HC exhibited robust context representations, while neither region demonstrated significant object representations (S11 Fig). These findings suggest a consistent involvement of the entire hippocampal axis in processing contextual information during spatial navigation.

### Object representations in EC

**Fig 3:**
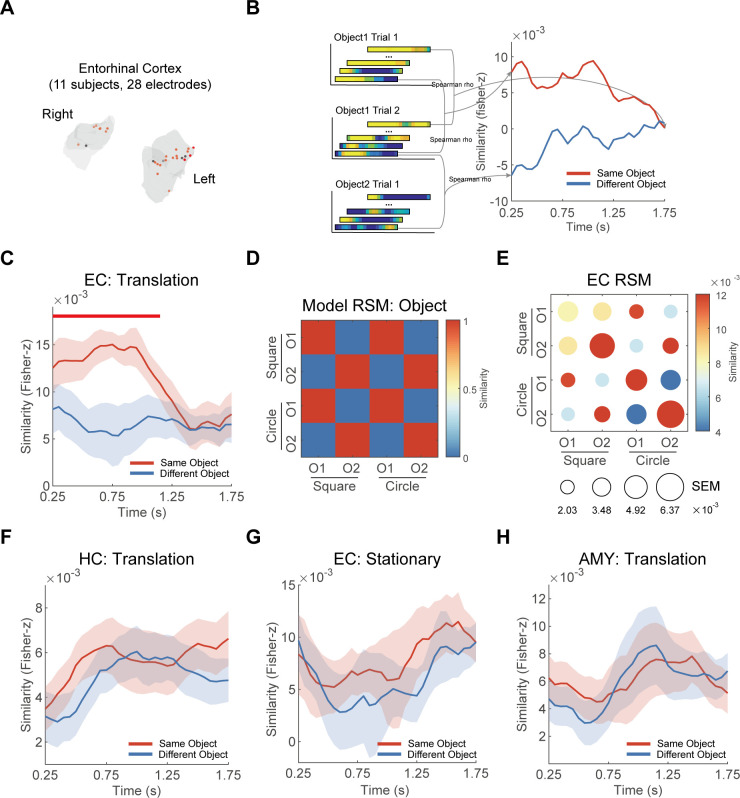
Object representations in EC. **(A)** The positions of EC electrodes projected onto the MNI152 template from all 11 participants. The red dots are task-selective electrodes (*N* = 23). The black dots are non-task-selective electrodes (*N* = 5). **(B)** A schematic illustration of EC object representations. **(C)** During the period from 0.25 to 1.15 s after the translation onset, the similarity of Same Object (the red line) was significantly greater compared to the similarity of Different Object (the blue line) in EC. The horizontal red line marked the time window where these significant differences were observed. **(D)** The representational similarity matrix (RSM) for object representation model. **(E)** Group-level RSM of EC. Circle color represents the mean representational similarity, while the size denotes SEM across participants. **(F)** During the translation epochs, HC did not exhibit object representation. **(G)** During the stationary epochs, EC did not show object representation. **(H)** During the translation epochs, the amygdala did not exhibit object representation. The shaded areas represent SEM across participants in panels **C, F, G, and H**.

We then investigated whether the EC represented objects during memory retrieval. For this analysis, we used the data from 23 EC task-selective electrodes from 10 participants (Fig 3A; see S1 Table for details). Consistent with our previous approach, we restricted our primary analysis to good trials (as detailed in the “Representational similarity analysis”).

Using RSA (Fig 3B), we found that EC activity showed significantly greater similarity for Same Object (red line in Fig 3C) compared to Different Object (blue line in Fig 3C) during translation epochs. This effect was observed between 0.25 s and 1.15 s after the onset of translation (*p*_cluster_ < 0.001). To visually illustrate the object representations in EC, we averaged the similarities across time windows with significant differences between Same Object and Different Object, creating a 4 × 4 RSM for each participant. Fig 3D shows the theoretical model of object representation, while Fig 3E presents the group-averaged RSM in EC. These results showed robust object representations in EC. However, we did not find significant object representations in HC (Fig 3F).

Furthermore, our analysis revealed the absence of object representations in EC during stationary epochs (Fig 3G). And no object representations were observed in the amygdala during the translation epochs (Fig 3H). Control analyses regarding trial types, electrode types, as well as parameters for the time window consistently demonstrated robust object representations in EC while confirming their absence in HC (S4–S10 Figs). For comparative purposes, we examined EC activity during the translation epochs of bad trials. Our analysis demonstrated no significant context or object representations within EC in these trials (S13C–S13D Fig). Furthermore, a direct comparison of EC object representation strength between good and bad trials did not yield a statistically significant difference (all *p* values > 0.05), consistent with the pattern observed in hippocampal context representations and likely reflecting similar constraints of high performance and limited trial number. These findings provide strong evidence for the functional specialization of EC in object processing during spatial navigation tasks.

### Coordination of HC context and EC object representations supports spatial navigation

**Fig 4:**
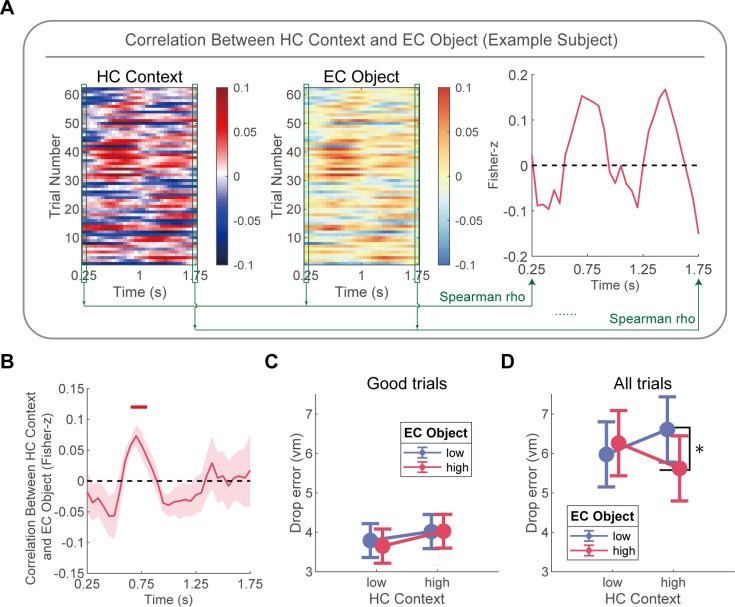
Coordination of HC context and EC object representations. **(A)** For an example subject, we adopted a leave-one-trial-out approach to obtain trial-level HC context representation strength (the left panel) and EC object (the middle panel) representation strength within each time window. Specifically, for each trial and time window, HC context strength was calculated by subtracting its average correlation with trials of a different context from its average correlation with trials of the same context; EC object strength was similarly calculated by subtracting its average correlation with trials of different objects from its average correlation with trials of the same object, iterated across all trials. We then calculated the Spearman correlation between the derived HC context representation strength and EC object representation strength across trials for each corresponding time window (the green boxes and lines). **(B)** There was a significant positive correlation between HC context and EC object representations within the time window of 0.65–0.80 s after the onset of translation. The horizontal red line indicates the significant time window. **(C)** Neural representations did not significantly modulate behavioral performance in good trials. **(D)** There was a significant interaction between HC context and EC object representations in all trials. Specifically, in trials with high context representation, stronger object representation led to a reduction in drop error, whereas this effect was absent in trials with low context representation. The shaded areas represent SEM across participants in panel **B**. The error bars in panels **C and D** represent the SEM estimated by LMEs. The data underlying this figure can be found in https://doi.org/10.5281/zenodo.17017876.

To assess the consistency of HC context and EC object representations, we employed a leave-one-trial-out approach to obtain trial-level strengths of HC context and EC object representations (see “Coordinated analysis of HC context and EC object representations” for details). Then, we calculated the Spearman correlation between the strengths of HC context and EC object representations across trials within individual participant, analyzing each time window separately (Fig 4A). For this analysis, we included the data from 10 participants with task-selective electrodes in both HC and EC (see S1 Table for details). And we maintained the same temporal parameters used in the previous RSA: a 500 ms sliding window width with 50 ms step size and 90% overlap. Our results indicated a significant positive correlation between HC context and EC object representations in the time window of 0.65–0.80 s after translation onset (*p*_cluster_ = 0.028, Fig 4B) for good trials but not for bad trials (S23A Fig). Further lagged correlation analysis revealed a peak correlation between HC and EC representations at HC time = 0.65 s and EC time = 0.85 s (S21B Fig; Fisher-*z* = 0.0891, *t*_9_ = 2.868, *p* = 0.0093). Subsequent comparison of mean correlation values demonstrated significantly stronger correlations for HC-leading-EC time-window pairs than for EC-leading-HC pairs (S21D Fig; *t*_9_ = 2.169, *p* = 0.029). This finding suggests a coordinated relationship between HC context and object representations during the critical time window, prompting us to further investigate how these neural representations jointly influence behavioral performance.

We examined the relationship between behavioral performance and neural representations using a two-level linear mixed-effects (LME) model, with trials nested within subjects. For each trial, we calculated trial-level strengths of HC context and EC object representations by averaging the representations within the 0.65–0.80 s time window following translation onset. Trials were then categorized into “high” or “low” groups for both HC context representation (factor 1: Context Representation) and EC object representation (factor 2: Object Representation) based on whether their respective strengths exceeded the within-subject median. We constructed an LME model with these two factors and their interaction as fixed effects, drop error as the dependent variable, and subject number as a random effect.

Initial analysis of good trials revealed no significant modulatory effect of neural representations on behavioral performance (Fig 4C). We speculated that the high levels of both behavioral performance and neural representations in good trials might have obscured potential relationships between these measures. To address this, we repeated the analysis across all trials, which allowed for a more comprehensive examination of the relationship. The results indicated a significant interaction between Context Representation and Object Representation (*F*_(1, 860.79)_ = 3.996, *p* = 0.0459). The main effects of Context Representation (*F*_(1, 860)_ = 0.0002, *p* = 0.9897) and Object Representation (*F*_(1, 860)_ = 1.23, *p* = 0.2677) were not significant. Simple effects analysis indicated that drop error was significantly lower only when both the context representation and the object representation were strong (Fig 4D). Specifically, in trials with high context representation, stronger object representation was associated with reduced drop error (*t*_860_ = −2.193, *p* = 0.0285), whereas no such effect was observed in trials with low context representation (*t*_860_ = 0.637, *p* = 0.5243). These results suggest that the modulation of behavioral performance by object representation in EC is contingent on the strength of context representation in HC, highlighting the importance of their coordinated activity in driving successful navigation.

### Low-frequency dependence of HC context and EC object representations

Consistent with previous findings highlighting the importance of low-frequency activity in the medial temporal lobe for spatial navigation [21–24], we hypothesized that HC context and EC object representations may be mainly contributed by low-frequency activity (2–8 Hz) rather than high-frequency activities (8–30 Hz and 30–150 Hz). To test this hypothesis, we adapted established methodologies [14,15] and use a jackknife method to identify frequency-specific contributions (see “Frequency-specific analysis and jackknife procedure” for details). In brief, we removed the power from one of three frequency bands when calculating representational similarity, creating the difference of representational similarity without particular frequency band information. We then subtract this difference from the similarity difference obtained using the full frequency band, yielding the representation reduction. Finally, we examined whether the representation reduction was significantly greater than zero. A significant representation reduction implies that the activity of that removed frequency band significantly contributes to the representation.

**Fig 5:**
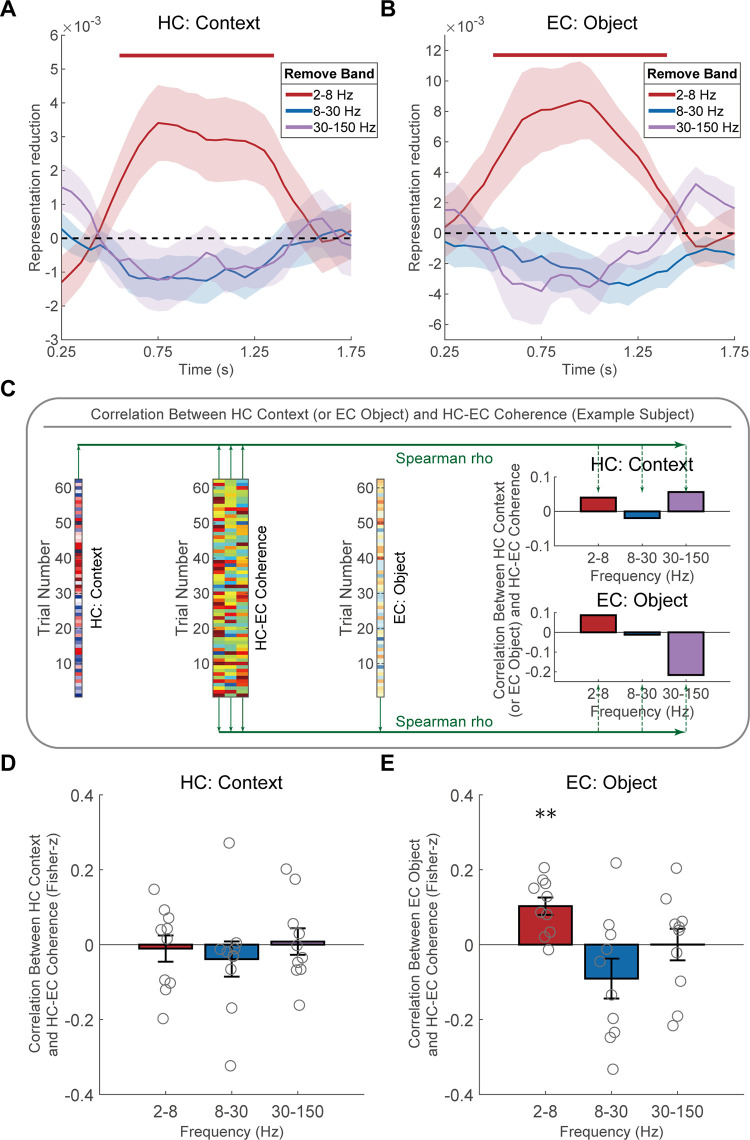
Low-frequency dependence of HC context and EC object representations. **(A)** Removal of low-frequency (2–8 Hz) activity from the broadband activity significantly reduced HC context representation. The horizontal red line indicated the time window that was significantly greater than zero. While there was no significant decrease of HC context representation after excluding 8–30 Hz or 30–150 Hz activity. **(B)** Removal of low-frequency (2–8 Hz) activity from the broadband activity significantly reduced EC object representation. The horizontal red line indicated the time window that was significantly greater than zero. While there was no significant decrease of EC object representation after excluding 8–30 Hz or 30–150 Hz activity. **(C)** The same subject as in Fig 4A was used for demonstration. First, the matrices of HC context representation strength and EC object representation strength (of dimensions [trials × time windows]) were averaged across the time windows of HC–EC co-activation (Fig 4B, 0.65–0.80 s). This yielded corresponding vectors of HC context representation strength (the leftmost panel) and EC object representation strength (the right-of-center panel, of dimensions [trials × 1]). Subsequently, Spearman correlation was computed between each of these vectors and the trial-level coherence within each of three frequency bands (the left-of-center panel), yielding three correlation coefficients (rho) for each representation (the rightmost two panels). **(D)** HC context representation did not show significant correlations with functional connectivity between HC and EC in any frequency bands. **(E)** EC object representation exhibited a significant positive correlation with HC–EC functional connectivity in low-frequency band (2–8 Hz), whereas there was no significant correlation in 8–30 Hz and 30–150 Hz. ** *p* < 0.01. The shaded areas represent SEM across participants in panels **A and B**. The hollow circles indicate individual participant, and error bars indicate SEM across participants in panels **D and E**. The data underlying this figure can be found in https://doi.org/10.5281/zenodo.17017876.

We found that removing the low-frequency activity (2–8 Hz) led to significant reductions in both HC context (*p*_cluster_ = 0.002, Fig 5A) and EC object representations (*p*_cluster_ = 0.003, Fig 5B). Conversely, removing 8–30 Hz or 30–150 Hz activities did not result in significant reductions in HC context or EC object representations (Fig 5A and 5B). Extending RSA to include 0.1–2 Hz power confirmed the importance of 2–8 Hz power. Context/object-specific representations remained significant across the full bandwidth (0.1–150 Hz) while showing no dependence on very low-frequency (0.1–2 Hz) activity (S14 Fig). Critically, removal of the 0.1–2 Hz component from full-band signals did not diminish these effects, confirming their spectral independence from slower oscillations. Selective removal of the 2–8 Hz band, however, significantly attenuated context/object-specific representations. Moreover, when we reconstructed HC and EC representational patterns exclusively using activity in 2–8 Hz, context- and object-specific representations were robustly sustained (S22 Fig). We further conducted direct comparisons of mean low-frequency power in HC between the two environments during translation epochs. The results revealed no significant differences in hippocampal low-frequency power across environments (S20 Fig), effectively ruling out the possibility that context representation differences stemmed from variations in average power levels. These results suggest that the HC context and EC object representations are primarily driven by the low-frequency activity. To exclude the potential confound of non-oscillatory activity, we separated periodic and aperiodic components of the signal using a robust regression approach applied to the log-log transformed power spectrum [25,26]. After isolating oscillatory power in the 2–8 Hz band, we repeated the RSA and found that both hippocampal context representations and entorhinal object representations remained significant (S25 Fig). This confirms that our core results are driven by oscillatory activity rather than non-oscillatory spectral components.

Considering the coactivation of HC and EC representations and their dependence on low-frequency activity, we hypothesized that HC and EC communicated their representations through the low-frequency functional connectivity. From the raw signals of HC and EC, we first observed that there were obvious low-frequency activities during the translation movement (S12 Fig). We employed a novel correlation-based metric to explore the relationship between HC context and EC object representations and HC–EC functional connectivity, rather than using standard coherence alone (see “Coherence analysis” for details). We calculated the cross-time imaginary coherence between HC and EC during the translation epochs and transformed it into *z*-scores. Then, we calculated the correlations between the coherence and HC context and EC object representation across trials within each participant (Fig 5C). The *z*-scored imaginary coherence was averaged over the three previously mentioned frequency bands (i.e., 2−8 Hz, 8−30 Hz, 30−150 Hz). The strength of HC context and EC object representations were averaged over the 0.65–0.80 s time window after translation onset, as the previous result (Fig 4B) indicated significant positive correlation across trials within those time windows.

Our results revealed distinct patterns of correlation between neural representations and HC–EC coherence across frequency bands. Specifically, there was no significant correlation between HC context representations and HC–EC coherence in any frequency bands for good trials (Fig 5D, all *p* values > 0.05). In contrast, EC object representations showed a significant positive correlation with low-frequency (2–8 Hz) HC–EC coherence (Fig 5E; *t*_9_ = 4.453, *p* = 0.0024, FDR corrected), but not for high-frequency coherence for good trials (Fig 5E; 8–30 Hz, *t*_9_ = −1.70, *p* = 0.938; 30–150 Hz, *t*_9_ = 0.007, *p* = 0.746; FDR corrected). In contrast, neither HC context (S21B Fig) nor EC object (S21C Fig) representation showed significant correlations with HC–EC coherence in any frequency band for bad trials. Together, these results highlight the importance of low-frequency neural dynamics in mediating both local representations and interregional interactions within the medial temporal lobe during spatial navigation.

## Discussion

In this study, we investigated the distinct functional contributions of the human hippocampal–entorhinal circuit to context-dependent spatial memory retrieval, with particular emphasis on interregional coupling during the reactivation of spatial representations. Our findings revealed a clear dissociation in information processing between these regions: contextual information reactivation was predominantly observed in the HC during translational movement, while object-related information reactivation was primarily detected in the EC. Crucially, we found that accurate spatial memory retrieval, as indicated by reduced drop errors, only occurred when both hippocampal contextual representations and entorhinal object representations were strongly activated, suggesting that their coordinated covariation was crucial for successful memory retrieval. Furthermore, our analysis of the relationship between these two types of representations revealed a significant positive correlation between low-frequency oscillatory synchronization in the HC–EC circuit and object information reactivation in the EC. These findings collectively suggest that low-frequency oscillations play a pivotal role in coordinating hippocampal–entorhinal interactions to support context-dependent spatial memory retrieval.

Our findings revealed distinct oscillatory patterns representing contextual and object information, demonstrating that mesoscopic-level LFP signals contain rich spatial information. This provides a valuable complement to existing human neuroimaging studies [5,7]. Given the highly dynamic nature of memory processes, LFP signals offer a unique opportunity to uncover the spatiotemporal characteristics of neural representations at a finer temporal scale. One prevailing theory proposes that hippocampal context representations exhibit gradient and hierarchical organization along the longitudinal axis. According to this view, anterior hippocampal representations are coarser and more schema-like, while posterior hippocampal representations are more precise and detail-oriented [20,27–29]. While this model suggests that context representations are primarily driven by anterior hippocampal activity, our findings demonstrate robust context representations in both anterior and posterior hippocampal regions. This observation aligns with recent studies. For instance, a iEEG study found that the posterior HC showed the reinstatement of item-context associations (see their S4 Fig) [14]. In an fMRI study with the similar paradigm to ours, researchers found that the multivoxel activity patterns in the posterior HC differentiated two environments (see their Fig 3) [5]. One possible reason why there emerged homogenous context representations in HC could be related to the task difficulty, since participants did not need to distinguish among multiple specific locations when navigating only two objects per environment. In spatial navigation, the gradient representation hypothesis of HC is primarily supported by experimental evidence that shows the difference in the size of place fields of rodent place cells along the anterior-posterior axis of HC, with dorsal place fields being smaller [20,29]. Our findings imply a potentially more intricate hippocampal function in humans, necessitating further research to assess cross-species validity of the hypothesis. In the previous studies, object representations are usually characterized by spectral patterns distributed widely across cortical electrodes, lacking precise localization [15,30,31]. As a primary output region of HC, EC was found showing object representations, expanding these findings and suggesting that previously identified representations might be the result of EC coordination with a more broadly distributed neocortical network. Recent single neuron studies in rodents and humans have also identified object cells in EC [8,32], which may underlie the object-related oscillatory representations observed in our study. The presence of such specialized neural populations may provide the cellular basis for the object-related oscillatory representations observed in our LFP recordings, suggesting a potential mechanism by which object information is processed and integrated within the medial temporal lobe memory system.

Neural representations in the brain must not only reliably encode information about the external world but also deliver this information in a manner that effectively guides behavior. In our study, we demonstrated that HC context representations are temporally coupled with EC object representations within a specific transient time window. Crucially, we found that accurate spatial memory performance only occurred when both hippocampal context representations and entorhinal object representations showed strong concurrent activation. This finding highlights the essential role of their coordinated coactivation in successful target memory retrieval. These results align with and extend previous findings across different methodologies and species. Rodent study has shown increased oscillatory coherence between the hippocampus and entorhinal cortex preceding successful odor-location association recognition, although this study did not specifically identify the reactivation of odor or location representations during this process [33]. Human neuroimaging research has demonstrated that the covariation of hippocampal context representations and entorhinal grid-like representations predict behavioral performance [5]. Our findings significantly extend this understanding by revealing the critical importance of object representations in this process. Although these approaches utilize different signal modalities, both lines of research converge on the conclusion that successful memory retrieval requires the coordinated reactivation of different components of episodic memory. This conclusion is further supported by a recent human single-neuron study, which found that hippocampal ripples are accompanied by the coactivation of object-responsive cells (encoding target objects) and place cells (encoding target locations) [32].

Rodent [34] and human MEG [35] studies suggest that hippocampal theta oscillations mediate the context-dependent retrieval of episodic memory. Our results extend these studies by the evidence that the hippocampal broadband activity during spatial navigation facilitate the distinction between two environments, primarily contributed by low-frequency activity, serving as a proxy of remapping of place cells. Theorists propose remapping as a fundamental mechanism for the brain’s high memory capacity, formalizing the hypothesis of compositional computation [12]. The theory predicts that when memories are retrieved based on contextual cues, hippocampal–entorhinal interactions should convey information about contexts, locations, and target objects to support the reconstruction of maps. In line with this proposal, we found that contextual information was reactivated within approximately 650 ms after movement onset, consistent with previous findings from word-image pairing paradigms [36]. Importantly, we discovered that low-frequency oscillatory synchronization between the HC and EC was positively correlated with EC object reactivation, but not with HC context reactivation. This suggests that EC object representations were invariably accompanied by enhanced low-frequency functional connectivity between HC and EC. A human single neuron recording study of word-image pairs found that firing rate of hippocampal neuron within a brief window (500–1,000 ms) after the cue onset was associated with entorhinal reactivation patterns [37]. Together, these studies suggest that brief oscillatory coupling could serve as a potential mechanism not only for connecting cell populations of HC and EC to accomplish memory retrieval, but also for information processing within each area.

The results of lagged correlation between HC context and EC object representations indicate that HC context representations temporally precede EC object representations (S21 Fig). This finding aligns with results in Fig 5D–5E, where EC object representations correlated with low-frequency HC–EC connectivity, whereas HC context representations showed no such relationship. Furthermore, Fig 4D demonstrates that behavioral modulation by EC object representations is contingent on HC context representation strength. These findings collectively suggest that during context-dependent memory retrieval: (1) context representations initially emerge in HC, (2) this representation engages subsequently emerging EC object representations via low-frequency HC–EC functional connectivity, and (3) this coupled mechanism supports successful context-dependent navigation. Furthermore, these results align with two previous human iEEG studies. Staresina and colleagues demonstrated that during associative memory retrieval, target objects can be successfully decoded from population-level spiking activity in EC, with hippocampal spiking patterns preceding EC activity and predicting the precision of object reinstatement in EC [37]. Complementarily, Pacheco Estefan and colleagues reported a spatiotemporal double dissociation in representational reinstatement: contextual associations were initially reinstated in HC, followed by later reinstatement of item information in lateral temporal cortex [14].

The selective emergence of HC context and EC object representations during translation aligns with the role of locomotion in activating MTL circuits. Isolating translation periods in our RSA analysis controls for confounding movement-related neural activity (e.g., motor planning and arousal level) and ensures that observed differences can reflect mnemonic processing. Furthermore, converging evidence indicates that active translation enhances MTL activation and modulates spatial coding [22,38–43], suggesting it provides an optimal neurophysiological state for processing task-relevant information.

While our paradigm established hippocampal context discrimination—aligning with foundational rodent studies demonstrating remapping across distinct environments [4,44–46]—the use of only two contexts restricts investigation into the representational capacity of contextual coding. Computational models propose that hippocampal attractor networks support high-capacity context representations [47,48], a feature empirically validated in rodents using multi-context designs (>10 environments) [49,50]. Future human intracranial studies employing high-density electrodes should test whether similar scalable coding exists in humans, advancing our understanding of episodic memory architecture.

In summary, this study provides new insights into the role of low-frequency oscillations in context-dependent spatial navigation. We demonstrate that the HC and EC exhibit distinct functional specializations, with the HC encoding contextual information and the EC processing object-related information, both primarily mediated by low-frequency oscillatory activity. Crucially, we found that behavioral performance during spatial navigation tasks is predicted by the co-activation of oscillatory patterns in both the HC and EC, rather than by the pattern of either region alone. This finding underscores the importance of coordinated neural representations between these medial temporal lobe structures for successful spatial memory retrieval. Moreover, synchronization of these low-frequency oscillations between the HC and EC facilitates object representation in the EC. These findings offer a deeper understanding of the neural mechanisms underlying context-dependent memory retrieval.

## Methods

### Ethics statement

Written informed consent was obtained from all patients voluntarily prior to their inclusion in this study. The experimental protocol has been approved by the Institutional Review Board of Beijing Tiantan Hospital, Capital Medical University (KY2023-034-02).

### Participants

We recruited 31 epilepsy patients (10 females, 28 right-handed, mean age ± standard deviation (STD): 25.5 ± 6.3 years) who were implanted with stereotactic electrodes to locate their seizure foci to guide subsequent treatment. Electrode placement was determined solely based on clinical requirements. Participants were recruited from Beijing Tiantan Hospital, Capital Medical University. All had normal or corrected-to-normal vision.

### Experimental task

The experimental task was developed using Vizard 5.9 (WorldViz, Santa Barbara, CA) and executed on a Dell laptop with a 14-inch screen and 60 Hz refresh rate. Participants were required to learn and memorize the positions of two distinct objects within two virtual arenas. As shown in Fig 1A and 1B, each arena consisted of green grass, a wall, blue sky, and four different trees outside the wall. The two environments differed in their wall configurations: one with a square layout (29 vm side length, referred to as Square) and the other with a circular layout (29 vm diameter referred to as Circle). Walls were 2.3 virtual meter (vm) tall. Environment design incorporated shared visual elements to minimize low-level discriminability (see S1 Video). The view height was fixed at 1.6 vm. Movement accelerated rapidly to a fixed maximum speed of 3 vm/s. Navigation controls were customized for handedness: right-handed participants used arrow keys (up for forward movement, left/right for rotation), while left-handed participants used the W (forward), A (left rotation), and D (right rotation) keys.

The center of the environment was at the coordinate [0, 0]. The experiment consisted of two sessions. In each session, two objects in Square and Circle environment were the same, but placed in different locations. In session 1, the objects were a traffic cone and a pumpkin. In the Square environment, the traffic cone was placed at [4, 9] and the pumpkin at [−10, −5]. In the Circle environment, the traffic cone was placed at [−4, 9] and the pumpkin at [10, −5]. In session 2, the objects were a lamp and a plant. In the Square environment, the lamp was placed at [−2, 5] and the plant at [7, −4]. In the Circle environment, the lamp was placed at [2, 5] and the plant at [−7, −4]. Each session included a learning block and a test block, as shown in Fig 1B.

For trials in the learning block, participants first viewed a fixation point (Fixation), followed by a 2-s overhead view (Overlook) of the current trial’s environment devoid of objects. They then judged whether the wall shape was square or circle (Question). Subsequently, participants viewed the object picture (Cue) to be learned, followed by its appearance in the 3D environment. They then started from a random position and navigated to the object in a first-person perspective (Collect).

For trials in the test block, the procedure mirrored that of the learning trials until the object picture was shown. After presenting the object picture, the object did not appear in the environment, and the participants needed to walk to the location of the object in their memory and press the space key to respond (Replace). Then, participants received instantaneous visual feedback at the placement location via graded emoticons reflecting positional accuracy (Feedback): dark green smiley face (≤3 vm), light green smiley face (3–6 vm), yellow neutral face (6–9 vm), light red frowning face (9–12 vm), or dark red frowning face (>12 vm). Finally, the object appeared in its correct position, the participants need to navigating to the position of the object, starting from a random location (Recollect).

Each session comprised 20 learning trials and 50 test trials. Participants alternated environments after completing 3–5 consecutive trials in one, ensuring a consistent total across two environments. All participants followed the same trial sequence. Across the experiment, 140 predefined random starting locations were used (40 for learning trials, 100 for test trials), identical for all participants. For each trial, the starting orientation was independently randomized per participant and not shared across subjects. To preclude potential biases arising from participants’ visual preferences for distinct tree appearances (serving as distal landmarks) across environments, we randomly swapped tree visual appearances between environments for a subset of participants while maintaining identical spatial positions in a subset of participants (S1 Video).

### Behavioral data analysis

Behavioral performance was measured using the drop error, defined as the Euclidean distance between the position where the participant placed the object and the correct position of the object. To assess randomness in participants’ performance, we created a surrogate distribution of drop errors. In each environment, we randomly selected 10,000 drop positions for each object, resulting in eight distributions of drop errors (2 sessions × 2 environments × 2 objects). Then, we randomly sampled one drop error from each of the eight distributions and averaged these values, and this process was repeated 10,000 times to derive the surrogate distribution of the mean drop error (Fig 1C). The fifth percentile of this distribution (9.87 vm) served as a critical threshold for determining significant deviation from chance level performance (*p* < 0.05). Trials with drop errors below this critical threshold were considered as good trials.

We compared the drop errors between two environments (Square and Circle) and between the two sessions (Session 1 and Session 2) using a 2 × 2 repeated measures ANOVA (Fig 1E). To verify whether there was a significant learning effect within each session, we divided the 50 trials of each session into two equal parts: the first 25 trials (H1) and the last 25 trials (H2). Subsequently, we conducted another 2 × 2 repeated measures ANOVA to analyze the effects of session (S1 and S2) and time (H1 and H2) on drop errors (Fig 1F).

### Intracranial EEG recordings and preprocessing

Intracranial EEG data were recorded by a Nihon-Kohden system with a sampling rate of 2,000 Hz. Electrode localization was performed through co-registration of pre-implantation MRI and post-implantation CT scans (see “Electrode localization” for details). EEG data preprocessing was conducted using the EEGLAB toolbox [51] along with custom scripts. To speed up subsequent analysis, the raw data was downsampled to 500 Hz. Then, the original signal and power spectrum of each electrode contact were visually inspected to exclude abnormal electrodes. Among 5,106 electrode contacts across 31 participants, 27 contacts were excluded due to abnormal signals. After that, we implemented a white matter referencing approach. For each intracranial electrode, candidate reference contacts were chosen from contacts located in white matter, which was by visual inspection of the overlaid images. A single contact with little or no apparent EEG activity was finally chosen as the reference. To attenuate power line noise, notch filters were applied to the re-referenced signals at frequencies of 48–52 Hz, 98–102 Hz, and 148–152 Hz. Finally, we used two criteria to automatically detect artifacts. The time point that met one of the following conditions and the time window from 0.5 s before to 0.5 s after the artefact was counted as the artifact segment. First, the time point exceeded the original signal mean ± 4 STD; Second, time points where the envelope of the filtered signal (25–80 Hz) exceeded the mean ± 5 STD of the envelope [52]. These identified artifactual time points were marked for exclusion from further analysis.

### Electrode localization

Automatic parcellation of brain regions was performed on the patient’s pre-implantation T1 weighted structural images through the “*recon-all*” command in Freesurfer (v6.0.0, surfer.nmr.mgh.harvard.edu/). Then, post-implantation CT images were co-registered with the T1 images with the Statistical Parametric Mapping toolbox (SPM12, https://www.fil.ion.ucl.ac.uk/spm/). Finally, we used a toolbox to automatically localize and label intracranial electrodes, which used clustering-based segmentation and classified according to anatomical landmarks in native space [53]. For visualization of all patients’ electrodes on the average brain surface, each patient’s MRI was co-registered to MNI space. All electrodes were then superimposed onto the brain surface.

### Time–frequency analysis

To obtain a broadband power spectrum (for “Representational similarity analysis” and “Coherence analysis”), we took the following steps. First, we obtained the raw power for 50 frequency points, which were logarithmically spaced between 2 Hz and 150 Hz, using a wavelet convolution with a width of 5 cycles. Second, logarithmic transformation was performed on the raw power, and we calculated the mean and STD of the power across the full task duration (artifact-free periods) at each frequency. Third, the power values of each frequency point were z-scored using the mean value and STD obtained previously.

### Representational similarity analysis

To quantify the neural representations of environment and object during memory retrieval (i.e., the test block), we employed representational similarity analysis (RSA) [19]. Following previous studies [14,18,30,54], we determined the similarity between trials (epochs) by calculating the Spearman correlation of broadband power within a sliding time window of 500 ms across multiple electrodes in each region of interest (ROI). The ROIs we focused on were the hippocampus (HC) and the entorhinal cortex (EC), with the amygdala (AMY) serving as a control region. The specific procedures involved were as follows.

Firstly, we selected the longest translation movement period for each test trial, ensuring that it was no shorter than 1 s. Subsequently, we defined a 2-s analysis window starting from the onset of translation, referred to as translation epochs. We identified an average of 82.1 ± 14.0 translation epochs from 100 test trials (64.8 ± 15.2 for good trials). We excluded (mean ± std) 2 ± 3.25, 3.64 ± 6.29, and 5.71 ± 7.57 artifact-contaminated translation epochs in HC, EC, and AMY, respectively. Next, to reduce noise and get more stable representations, we excluded electrodes that did not differentiate between environments or objects on the raw signal [17]. Electrodes were classified as task-selective or non-task-selective based on preprocessed raw iEEG signals prior to time–frequency analysis. For each electrode, we extracted all 2-s translation epochs and segregated continuous voltage time series into 2 group by context labels (or object labels). We then performed two independent two-sample *t*-tests per electrode: (i) Context sensitivity test: Compared voltage distributions between different contexts; (ii) Object sensitivity test: Compared voltage distributions between different objects. Electrode inclusion required significant effects in both tests (conjunction threshold: *p* < 0.001 for both *t*-tests). Electrodes meeting this criterion were task-selective; others were excluded as non-task-selective. Across electrodes from all participants, 33 electrodes were removed from 188 HC electrodes, 5 from 28 EC electrodes, and 14 from 80 AMY electrodes (see S1 Table for details).

Secondly, as shown in Fig 2B, we applied wavelet convolution to the raw data of the translation epochs to obtain power at 50 frequency points with logarithmic equal spacing between 2 and 150 Hz, and standardized power to *z*-scores (see “Time–frequency analysis” for details). Then, each 2-s translation epoch was segmented into 31-time windows using a sliding window approach with a 500 ms width and 50 ms step size, allowing for overlap. The data within each window consisted of 50 × 250 × *N* dimensions (50 frequency points, 250 time points, *N* denotes the number of ROI electrodes included for each patient), which was then reshaped into a vector. Each epoch yielded a total of 31 vectors, with the central time of each window marked as the vector’s timestamp. Subsequently, we calculated the Spearman correlation coefficients (rho) between pairs of translation epochs, correlating the vectors corresponding to each time window within each pair individually. In the end, we applied Fisher’s *z*-transformation to the rho to obtain the *z* value.

Finally, for group-level analyses, we performed two contrasts. For context representations, we averaged *z*-values from epoch pairs within the same environment (Same Context) and across different environments (Different Context), respectively. We then performed one-tailed *t*-tests at the group level in each time window, with the assumption that *z*-value would be higher for Same Context compared to Different Context. Regarding object representations, we averaged *z*-values from epoch pairs with the same object label (Same Object) and different object labels (Different Object). Then we performed one-tailed *t*-tests at the group level in each time window, assuming that higher *z*-value for Same Object compared to Different Object. We employed cluster-based permutation tests for multiple comparison correction across time windows (see “Statistical analysis” for details). It was important to note that we conducted the above analyses separately within each session and then averaged the results from the two sessions.

To confirm robustness of our RSA results, we conducted a series of different control analyses. Firstly, for the data of stationary epochs, we repeated the above analysis with a slight variation: to obtain sufficient epochs, we set the duration criteria for stationary epochs to 0.5 s (resulting in 93.0 ± 15.7 stationary epochs, 72.4 ± 16.7 for good trials). We excluded (mean ± std) 2.72 ± 4.07, 6.09 ± 9.31, and 6.59 ± 9.36 artifact-contaminated stationary epochs in HC, EC, and AMY, respectively. Then we repeated the above analyses for the three ROIs (HC, EC, and AMY). Secondly, we repeated the aforementioned analyses across all electrodes within the ROI, including non-task-selective electrodes (S5–S6 Figs). Thirdly, we repeated the RSA based on different time windows used to construct the neural vectors. Specifically, the time window was changed from a width of 500 ms with a step size of 50 ms to a width of either 200 ms with a step size of 20 ms (S7–S8 Figs), or a width of 100 ms with a step size of 10 ms (S9–S10 Figs). Finally, we repeated the above analyses separately in all test trials and good test trials. All results were similar to those reported in the main text.

It should be noted that 71.14% of translation epochs maintained pure translation movement throughout the 2 s analysis window, while for stationary epochs, only 23.14% persisted purely stationary versus 76.86% transitioned to other movement states (primarily to rotation, S15 Fig). Crucially, when restricting analyses to continuous 2 s pure translation movement periods: core findings remained statistically significant for translation (S16 Fig) and all null effects were preserved for stationary epochs (S17 Fig), confirming the robustness of state-specific neural processes. However, given that subsequent cross-trial correlation analyses require sufficient trial number and primarily focus on translation epochs, we retained the original duration threshold in both the RSA results and follow-up analyses presented in the main text.

### Coordinated analysis of HC context and EC object representations

To evaluate the consistency in representation strength between HC context and EC object representations, we adopted a leave-one-trial-out approach to obtain their trial-level strengths. For example, for a square trial, we calculated its correlation with all other square trials (i.e., epoch) and averaged them to get the similarity of Same Context; then we calculated its correlation with all other circle trials and averaged them to get the similarity of Different Context. By subtracting the similarity of Different Context from the similarity of Same Context, we obtained the context representation strength for that trial. This procedure was applied iteratively across all trials and time windows. The calculation of EC object representation strength follows a similar procedure, with the distinction being that we categorized other trials based on the object identity label of the leave-out trial, grouping them into either trials with the same object or trials with different objects. All these processes were conducted within each session. For this analysis, we utilized data from 10 patients with task-selective electrodes in both HC and EC (see S1 Table for details).

Afterwards, we calculated the Spearman correlation between HC context representation strength and EC object representation strength across trials for each participant, for each corresponding time window. Finally, at the group level, we statistically tested whether these Fisher *z*-transformed correlation values were significantly greater than zero, and conducted a cluster-based permutation test correction for the time windows. Importantly, trials with drop errors larger than the chance level (9.87 vm) were excluded to ensure accurate representation strength estimation, leaving this analysis only for good trials.

To examine the relationship between behavioral performance and neural representations, we constructed a two-level linear mixed-effects (LME) model, with trials nested within subjects. First, for each trial, we averaged the HC context representation and EC object representation within the time window of 0.65–0.80 s after the onset of translation (during which the two representations exhibited significant cross-trial positive correlations), resulting in trial-level strengths for both representations. Next, trials were divided into “high context representation” and “low context representation” groups (factor $X_{1}$: Context Representation, two levels: high versus low) based on whether the HC context representation strength exceeded the within-subject median. Similarly, trials were divided into “high object representation” and “low object representation” groups (factor $X_{2}$: Object Representation, two levels: high versus low) based on whether the EC object representation strength exceeded the within-subject median. Finally, we built an LME model using the two factors mentioned above and their interaction as fixed effects, the drop error as the dependent variable (Y), and the subject number (grouping variable, SubID) as a random effect. The formula is as follows:


$$\begin{array}{c} Y\;\sim \;1+X_{1}+X_{2}+X_{1}*X_{2}+\left(1|SubID\right) \end{array}$$ (1)

We initially performed the above analysis on good trials but did not observe a significant modulatory effect of neural representations on behavioral performance. Considering that both behavioral performance and neural representations may have already reached high levels in good trials, this could potentially obscure the relationship between neural representations and behavioral performance (analogous to a ceiling effect). Therefore, we repeated the analysis across all trials.

### Frequency-specific analysis and jackknife procedure

To capture the frequency specificity of context representations in HC and object representations in EC, we applied a jackknife procedure [14,15] on frequency features. We focused on three frequency bands: 2–8 Hz (16 frequency points), 8–30 Hz (15 frequency points), and 30–150 Hz (19 frequency points). Previously, when calculating the representational similarity differences, we used broadband spectral frequency information ranging from 2 to 150 Hz, which we referred to as RSA_FullFreq_ (for HC context representations, it was the similarity of Same Context minus the similarity of Different Context; for EC object representations, it was the similarity of Same Object minus the similarity of Different Object). Then, we computed RSA_RemoveBand_ (where “RemoveBand” was one of the three frequency bands) by excluding data from one of the three frequency bands in the vector. We subtracted RSA_RemoveBand_ from RSA_FullFreq_ to obtain the representation reduction for each frequency band. Finally, we performed statistical test on this representation reduction. If the similarity reduction for a specific frequency band was significantly greater than zero, it indicated that the contribution of the removed frequency band to the representational similarity (RSA_FullFreq_) was significant.

### Coherence analysis

We calculated the connectivity between HC and EC during translation epochs. For this purpose, we computed the imaginary coherence over the 0–2 s time window relative to translation onset, which is defined by the following formula:


$$\begin{array}{c} {ImagC}_{xy}=\;\frac{\left|Imag\left(S_{xy}(f)\right)\right|}{S_{xx}(f)S_{yy}(f)} \end{array}$$ (2)

where, $S_{xy}(f)$ is the cross-spectral density between electrode *x* and electrode *y*, while $\left|Imag(S_{xy}(f))\right|$ indicates the magnitude of its imaginary component. $S_{xx}(f)$ and $S_{yy}(f)$ represent the power spectral densities of electrode *x* and *y*, respectively. We employed wavelet convolution to obtain the cross-spectral density and power spectral densities (with parameters specified in “Time–frequency analysis”). We calculated the cross-time imaginary coherence, denoted as ${ImagC}_{xy}$, for each translation epoch.

Next, we z-transformed ${ImagC}_{xy}$ to obtain ${ZImagC}_{xy}$. To do this, we randomly shuffled the trials of one electrode for 1000 times. After each shuffle, we recalculated ${ImagC}_{xy}$ to generate a surrogate distribution. We then subtracted the mean of the surrogate distribution from the real ${ImagC}_{xy}$ and divided it by the STD of the surrogate distribution to obtain the ${ZImagC}_{xy}$. We repeated this calculation for each pair of HC–EC electrodes in the ipsilateral hemisphere. Finally, we averaged the electrode pairs within the participant to obtain the ${ZImagC}_{xy}$ for all translation epochs for each participant. The above process was repeated across 50 frequency points (see “Time–frequency analysis”), and the ${ZImagC}_{xy}$ at these frequency points was averaged into three frequency bands: 2–8 Hz, 8–30 Hz, and 30–150 Hz.

To analyze the relationship between HC–EC connectivity and HC context representations and EC object representations, we computed Spearman correlation (rho) between ${ZImagC}_{xy}$ and the strength of HC context representation and EC object representation across trials within each participant, for each frequency band. It should be noted that for the representation strengths of HC and EC in each translation epoch, we only averaged the values within the time window of 0.65–0.80 s after the onset of translation movement, because the strengths of HC context and EC object representations showed a strong positive correlation across trials only in this time window. Then, we conducted a group-level *t* test to determine whether the Fisher *z*-transformed correlation coefficient was significantly greater than zero. For the above analyses, we utilized data from 10 participants with task-selective electrodes in both HC and EC (see S1 Table for details).

### Statistical analysis

Repeated-measure ANOVA was used for behavioral data analysis. For RSA, we used one-sample *t*-tests. Unless otherwise specified, one-sample *t*-tests were performed a priori using one-tailed tests with α = 0.05. Multiple comparisons correction (Fig 5C–5D) was performed through the Benjamini–Hochberg method [55] for false discovery rate (FDR) correction, which was carried out by MATLAB codes developed by others [56]. For the multiple comparison correction of time windows (Figs 2–3), we applied a cluster-based permutation test at the subject level [56,57]. In brief, we randomly shuffled the condition labels (Same Context versus Different Context and Same Object versus Different Object), then used the *t* test to obtain the largest continuous significant time windows, and summed *t* values in the significant range. The above process was repeated 1,000 times to obtain a surrogate distribution of the summed *t* values. Finally, we assessed whether the summed *t*-value of the real significant time windows points surpassed the 95th percentile of the surrogate distribution. For the multiple comparison correction of time windows (Figs 4–5), we employed a cluster-based non-parametric permutation test wherein temporal clusters were first identified through continuous time point-wise *t*-tests (*p* < 0.05 threshold) across subjects. A null distribution was then generated by performing 1,000 permutations with random sign-flipping of the subject-specific time series of Fisher *z*-transformed correlation coefficients (50% probability), from which the maximal cluster mass was extracted per permutation. Statistical significance was ultimately determined by comparing observed cluster masses against this null distribution [56,57]. The LME model was implemented using the *lmerTest* package [58] in *R*, and the *emmeans* package was used to analyze interaction effects and simple effects. The degree of freedom for the LME model was estimated using the Satterthwaite approximation.

## Supporting information

S1 Fig Drop positions over 31 participants. **(A)** Drop positions for the cone in the circle environment. **(B)** Drop positions for the pumpkin in the circle environment. **(C)** Drop positions for the cone in the square environment. **(D)** Drop positions for the pumpkin in the square environment. **(E)** Drop positions for the lamp in the circle environment. **(F)** Drop positions for the plant in the circle environment. **(G)** Drop positions for the lamp in the square environment. **(H)** Drop positions for the plant in the square environment. Blue circles in **A, B, E, and F** are the boundary of circle environment. Red squares in **C, D, G, and H** are the boundary of square environment. Solid green dots outside the boundaries indicate trees. Small red dots inside the boundaries are drop positions of good trials. Small gray dots inside the boundaries are drop positions of bad trials. Different color star represents different object. The red star represents the cone. The yellow star represents the pumpkin. The black star represents the lamp. The green star represents the plant. The dashed-line circles represent a radius of 9.87 vm centered on the current target object. Any trials placed the object within this area are deemed good trials.
(TIF)

S2 Fig Return trajectories toward the target location over 31 participants. **(A)** Trajectories toward the cone in the circle environment. **(B)** Trajectories toward the pumpkin in the circle environment. **(C)** Trajectories toward the cone in the square environment. **(D)** Trajectories toward the pumpkin in the square environment. **(E)** Trajectories toward the lamp in the circle environment. **(F)** Trajectories toward the plant in the circle environment. **(G)** Trajectories toward the lamp in the square environment. **(H)** Trajectories toward the plant in the square environment. Red lines are trajectories toward the target location for good trials. Gray lines are trajectories toward the target location for bad trials. Notably, the participants move more straight toward the target location for good trials.
(TIF)

S3 Fig Duration of return trajectory and translation epoch. **(A)** The distribution of durations during which participants place objects back in all test trials. **(B)** The distribution of durations during which participants place objects back in good trials. **(C)** The distribution of durations for all translation movement epochs during which participants place objects back in all test trials. **(D)** The distribution of durations for all translation movement epochs during which participants place objects back in good trials. **(E)** The distribution of durations for the longest translation movement epoch in the return period of each test trial. **(F)** The distribution of durations for the longest translation movement epoch in the return period of each good test trial. In each panel, the red line represents the mean of the distribution, and the blue line represents the median of the distribution.
(TIF)

S4 Fig RSA results calculated in 500 ms time windows for all test trials on task-selective electrodes. **(A)** For the translation epochs from all trials, the similarity of Same Context was significantly greater than the similarity of Different Context in HC. The horizontal red line marks the time window where these significant differences were observed. **(B)** For the translation epochs from all trials, there was no significant difference between the similarity of Same Object and the similarity of Different Object in HC. **(C)** For the translation epochs from all trials, there was no significant difference between the similarity of Same Context and the similarity of Different Context in EC. **(D)** For the translation epochs from all trials, the similarity of Same Object was significantly greater than the similarity of Different Object in EC. The horizontal red line indicated the time window where these significant differences were observed. The shaded areas represent SEM across participants.
(TIF)

S5 Fig RSA results calculated in 500 ms time windows for all test trials on all electrodes in the ROI. **(A)** For the translation epochs from all trials, the similarity of Same Context was significantly greater than the similarity of Different Context in HC. The horizontal red line marks the time window where these significant differences were observed. **(B)** For the translation epochs from all trials, there was no significant difference between the similarity of Same Object and the similarity of Different Object in HC. **(C)** For the translation epochs from all trials, there was no significant difference between the similarity of Same Context and the similarity of Different Context in EC. **(D)** For the translation epochs from all trials, the similarity of Same Object was significantly greater than the similarity of Different Object in EC. The horizontal red line indicated the time window where these significant differences were observed. The shaded areas represent SEM across participants.
(TIF)

S6 Fig RSA results calculated in 500 ms time windows for good trials on all electrodes in the ROI. **(A)** For the translation epochs from good trials, the similarity of Same Context was significantly greater than the similarity of Different Context in HC. The horizontal red line marks the time window where these significant differences were observed. **(B)** For the translation epochs from good trials, there was no significant difference between the similarity of Same Object and the similarity of Different Object in HC. **(C)** For the translation epochs from good trials, there was no significant difference between the similarity of Same Context and the similarity of Different Context in EC. **(D)** For the translation epochs from good trials, the similarity of Same Object was significantly greater than the similarity of Different Object in EC. The horizontal red line indicated the time window where these significant differences were observed. The shaded areas represent SEM across participants.
(TIF)

S7 Fig RSA results calculated in 200 ms time windows for all test trials on task-selective electrodes. **(A)** For the translation epochs from all trials, the similarity of Same Context was significantly greater than the similarity of Different Context in HC. The horizontal red line marks the time window where these significant differences were observed. **(B)** For the translation epochs from all trials, there was no significant difference between the similarity of Same Object and the similarity of Different Object in HC. **(C)** For the translation epochs from all trials, there was no significant difference between the similarity of Same Context and the similarity of Different Context in EC. **(D)** For the translation epochs from all trials, the similarity of Same Object was significantly greater than the similarity of Different Object in EC. The horizontal red line indicated the time window where these significant differences were observed. The shaded areas represent SEM across participants.
(TIF)

S8 Fig RSA results calculated in 200 ms time windows for good trials on task-selective electrodes. **(A)** For the translation epochs from good trials, the similarity of Same Context was significantly greater than the similarity of Different Context in HC. The horizontal red line marks the time window where these significant differences were observed. **(B)** For the translation epochs from good trials, there was no significant difference between the similarity of Same Object and the similarity of Different Object in HC. **(C)** For the translation epochs from good trials, there was no significant difference between the similarity of Same Context and the similarity of Different Context in EC. **(D)** For the translation epochs from good trials, the similarity of Same Object was significantly greater than the similarity of Different Object in EC. The horizontal red line indicated the time window where these significant differences were observed. The shaded areas represent SEM across participants.
(TIF)

S9 Fig RSA results calculated in 100 ms time windows for all test trials on task-selective electrodes. **(A)** For the translation epochs from all trials, the similarity of Same Context was significantly greater than the similarity of Different Context in HC. The horizontal red line marks the time window where these significant differences were observed. **(B)** For the translation epochs from all trials, there was no significant difference between the similarity of Same Object and the similarity of Different Object in HC. **(C)** For the translation epochs from all trials, there was no significant difference between the similarity of Same Context and the similarity of Different Context in EC. **(D)** For the translation epochs from all trials, the similarity of Same Object was significantly greater than the similarity of Different Object in EC. The horizontal red line indicated the time window where these significant differences were observed. The shaded areas represent SEM across participants.
(TIF)

S10 Fig RSA results calculated in 100 ms time windows for good trials on task-selective electrodes. **(A)** For the translation epochs from good trials, the similarity of Same Context was significantly greater than the similarity of Different Context in HC. The horizontal red line marks the time window where these significant differences were observed. **(B)** For the translation epochs from good trials, there was no significant difference between the similarity of Same Object and the similarity of Different Object in HC. **(C)** For the translation epochs from good trials, there was no significant difference between the similarity of Same Context and the similarity of Different Context in EC. **(D)** For the translation epochs from good trials, the similarity of Same Object was significantly greater than the similarity of Different Object in EC. The horizontal red line indicated the time window where these significant differences were observed. The shaded areas represent SEM across participants.
(TIF)

S11 Fig Both anterior HC and posterior HC show context representations. **(A)** For the activity in the anterior HC, the similarity of Same Context was significantly greater than the Different Context. The horizontal red line indicated the time window where these significant differences were observed. **(B)** For the activity in the anterior HC, there was no significant similarity difference between Same Object and Different Object. **(C)** For the activity in the posterior HC, the similarity of Same Context was significantly greater than Different Context. The horizontal red line marks the time window where these significant differences were observed. **(D)** For the activity in the posterior HC, there was no significant similarity difference between Same Object and Different Object. The shaded areas represent SEM across participants.
(TIF)

S12 Fig Raw data and filter low-frequency signal in HC and EC. **(A–D)** Raw signals (black lines) and filter low-frequency (2–8 Hz) signals (red lines) of four translation epochs from a HC electrode and one EC electrode of Sub06. **(E–H)** Raw signals (black lines) and filter low-frequency (2–8 Hz) signals (red lines) of four translation epochs from a HC electrode and one EC electrode of Sub15. **(I–L)** Raw signals (black lines) and filter low-frequency (2–8 Hz) signals (red lines) of four translation epochs from a HC electrode and one EC electrode of Sub22. **(M–P)** Raw signals (black lines) and filter low-frequency (2–8 Hz) signals (red lines) of four translation epochs from a HC electrode and one EC electrode of Sub27.
(TIF)

S13 Fig RSA results of bad trials. For the translation epochs from bad trials, neither HC nor EC showed significant differences in neural representational similarity between Same Context versus Different Context conditions **(A and C)** or between Same Object versus Different Object conditions **(B and D)**. The shaded areas represent SEM across participants.
(TIF)

S14 Fig RSA results across a broader frequency band (0.1–150 Hz). **(A)** For the translation epochs from good trials, the similarity of Same Context was significantly greater than the similarity of Different Context in HC. The horizontal red line marks the time window where these significant differences were observed. **(B)** For the translation epochs from good trials, there was no significant difference between the similarity of Same Object and the similarity of Different Object in HC. **(C)** For the translation epochs from good trials, there was no significant difference between the similarity of Same Context and the similarity of Different Context in EC. **(D)** For the translation epochs from good trials, the similarity of Same Object was significantly greater than the similarity of Different Object in EC. The horizontal red line indicated the time window where these significant differences were observed. **(E)** In broader-frequency-band RSA results, removal of 2–8 Hz activity significantly reduced HC context representation. The horizontal red line indicated the time window that was significantly greater than zero. However, removing activity in the 0.1–2 Hz, 8–30 Hz, or 30–150 Hz bands did not significantly reduce HC context representation. **(F)** In broader-frequency-band RSA results, removal of 2–8 Hz activity significantly reduced EC object representation. The horizontal red line indicated the time window that was significantly greater than zero. While there was no significant decrease of EC object representation after excluding 0.1–2 Hz, 8–30 Hz, or 30–150 Hz activity. The shaded areas represent SEM across participants.
(TIF)

S15 Fig Movement states of all translation (A) and stationary (B) epochs in All participants. Stationary refers to states with zero linear velocity and zero angular velocity. Rotation is defined by zero linear velocity and non-zero angular velocity. Translation refers to states with non-zero linear velocity and zero angular velocity. Turing motion is defined by non-zero linear velocity and non-zero angular velocity.
(TIF)

S16 Fig RSA results from translation epochs exceeding 2 s duration. **(A)** For the translation epochs from good trials, the similarity of Same Context was significantly greater than the similarity of Different Context in HC. The horizontal red line marks the time window where these significant differences were observed. **(B)** For the translation epochs from good trials, the similarity of Same Object was significantly greater than the similarity of Different Object in EC. The horizontal red line indicated the time window where these significant differences were observed. The shaded areas represent SEM across participants.
(TIF)

S17 Fig RSA results from stationary epochs exceeding 2 s duration. **(A)** For the stationary epochs from good trials, there was no significant difference between the similarity of Same Context and the similarity of Different Context in HC. **(B)** For the stationary epochs from good trials, there was no significant difference between the similarity of Same Object and the similarity of Different Object in EC. The shaded areas represent SEM across participants.
(TIF)

S18 Fig Absence of context representations in occipital and temporal electrodes. **(A)** The positions of temporal electrodes projected onto the MNI152 template from all 29 participants. The red dots are task-selective electrodes (*N* = 407). The black dots are non-task-selective electrodes (*N* = 107). **(B)** For the translation epochs from good trials, there was no significant difference between the similarity of Same Context and the similarity of Different Context in temporal cortex. **(C)** The positions of occipital electrodes projected onto the MNI152 template from all 10 participants. The red dots are task-selective electrodes (*N* = 76). The black dots are non-task-selective electrodes (*N* = 30). **(D)** For the translation epochs from good trials, there was no significant difference between the similarity of Same Context and the similarity of Different Context in occipital cortex. The shaded areas represent SEM across participants in panels **B and D**.
(TIF)

S19 Fig Drop error of incorrect context-selection trials. **(A)** Number of correct and incorrect context-selection trials for each participant. **(B)** Among participants with context-selection errors (*N* = 21), no significant difference was observed in mean drop error between correct and incorrect context-selection trials. N.s. denotes “non-significant”. Hollow dots are individual subjects, and error bars indicate SEM across participants. **(C)** Given the unequal number of correct and incorrect context-selection trials, we randomly selected a matched number of correct trials per participant (equal to their error trials), averaged their drop errors, and performed participant-level paired *t*-tests. This procedure was repeated 10,000 times, with approximately 196 iterations yielding *t*-values exceeding the critical threshold (two-tailed paired *t* test, *df* = 20, *α* = 0.05, *t*_critical _= ±2.086, the red vertical lines, *p*_subsample_ = 1–196/10000 = 0.9804).
(TIF)

S20 Fig Hippocampal theta power in the square and circle environment. **(A)** *Z*-scored hippocampal theta power (2−8 Hz,) was significantly greater than zero during translation epochs from good trials in both the square (*t*_24_ = 2.279, *p* = 0.016) and circle (*t*_24_ = 3.309, *p* = 0.001) environments; furthermore, no significant difference emerged between environmental conditions (*t*_24_ = −0.911, *p* = 0.371). Hollow dots are individual subjects, and error bars indicate SEM across participants. N.s. denotes “non-significant”. * *p* < 0.05, ** *p* < 0.01.
(TIF)

S21 Fig Temporal relationship between HC context and EC object representations. **(A)** Data from one example subject. Trial-wise time-resolved representations of HC context (the left panel, of dimensions [trials × time windows]) and EC object (the middle panel, of dimensions [trials × time windows]), along with their time × time correlation matrix (the right panel, of dimensions [time windows × time windows]), computed using Spearman rho across trials. Warm colors in the matrix indicate positive correlations between HC and EC representation strength at corresponding time windows. **(B)** Group-level average of the time × time correlation matrix shown in **(A)**. The black solid lines highlight time window pairs of significant co-activation between HC context and EC object representations. The dashed line denotes the identity line (no time lag). The white star marks the peak correlation. **(C)** Time-lag map corresponds to **(B)**. Co-activation of HC and EC representations observed in blue regions denotes that EC representations precede HC representations. Conversely, co-activation observed in red regions indicates that HC representations precede EC representations. **(D)** An across-subject comparison of Fisher *z*-transformed peak correlation values in EC-leads-HC versus HC-leads-EC regions revealed significantly stronger co-activation when HC led EC. * *p* < 0.05.
(TIF)

S22 Fig RSA results based on theta power. **(A)** For the translation epochs from good trials, the similarity of Same Context was significantly greater than the similarity of Different Context in HC. The horizontal red line marks the time window where these significant differences were observed. **(B)** For the translation epochs from good trials, there was no significant difference between the similarity of Same Object and the similarity of Different Object in HC. **(C)** For the translation epochs from good trials, there was no significant difference between the similarity of Same Context and the similarity of Different Context in EC. **(D)** For the translation epochs from good trials, the similarity of Same Object was significantly greater than the similarity of Different Object in EC. The horizontal red line indicated the time window where these significant differences were observed. The shaded areas represent SEM across participants.
(TIF)

S23 Fig Coordinated HC–EC representations and coherence in bad trials. **(A)** No significant positive correlation was observed between HC context representation and EC object representation in bad trials. **(B)** In bad trials, HC context representation did not show significant correlations with functional connectivity between HC and EC in any frequency bands. **(C)** In bad trials, EC object representation did not show significant correlations with functional connectivity between HC and EC in any frequency bands. The shaded areas represent SEM across participants in panel **A**. The hollow circles indicate individual participant, and error bars indicate SEM across participants in panels **B and C**.
(TIF)

S24 Fig RSA results from the 10 participants with simultaneous hippocampal and entorhinal electrodes recordings. **(A)** The similarity of Same Context (the red line) was significantly greater compared to the similarity of Different Context (the blue line) in HC. The horizontal black line marked the time window where these significant differences were observed before multiple comparison correction (*p*_cluster_ = 0.13). **(B)** During the translation epochs, HC did not exhibit object representation. The shaded areas represent SEM across participants.
(TIF)

S25 Fig RSA results of oscillatory theta power after remove non-oscillatory activity. **(A)** We computed the power spectrum of translation epoch and transformed it into log–log space using the natural logarithm (the black line). A line was then fit to this spectrum in log–log space using a robust regression (the red line). This fitted line was transformed back into linear space to derive the aperiodic component of the signal, which was subsequently subtracted from the original spectrogram to isolate the periodic (oscillatory) power. Finally, we repeated our core RSA using this purified 2–8 Hz oscillatory power. **(B)** After remove non-oscillatory activity, theta power (2–8 Hz) in HC still represents the context information (cluster 1: 0.65–1 s, *p*_*cluster*_ = 0.065; cluster 2: 1.15–1.25 s, *p*_*cluster*_ = 0.185). The horizontal black lines mark the time windows showing a marginal significance, as identified by cluster-based permutation test. **(C)** After remove non-oscillatory activity, theta power (2–8 Hz) in EC still represents the object information (*p*_*cluster*_ = 0.001). The horizontal red line marks the time window identified by the cluster-based permutation test. The shaded areas represent SEM across participants in panels **B and C**.
(TIF)

S1 Table Electrode number. (DOCX)

S1 Video Example trials. (MP4)

## Abbreviations

EC: entorhinal cortex
FDR: false discovery rate
HC: hippocampus cortex
iEEG: intracranial electroencephalography
LFP: local field potential
LME: linear mixed-effects
ROI: regions of interest
RSA: representational similarity analysis
RSM: representational similarity matrix
