# Supplementary figures and images for: Coordinated hippocampal–entorhinal representations support human context-dependent spatial navigation
Source: PLoS Biol. 2025 Sep 17;23(9):e3003398. doi: 10.1371/journal.pbio.3003398 (PMC12453184; doi:10.1371/journal.pbio.3003398)

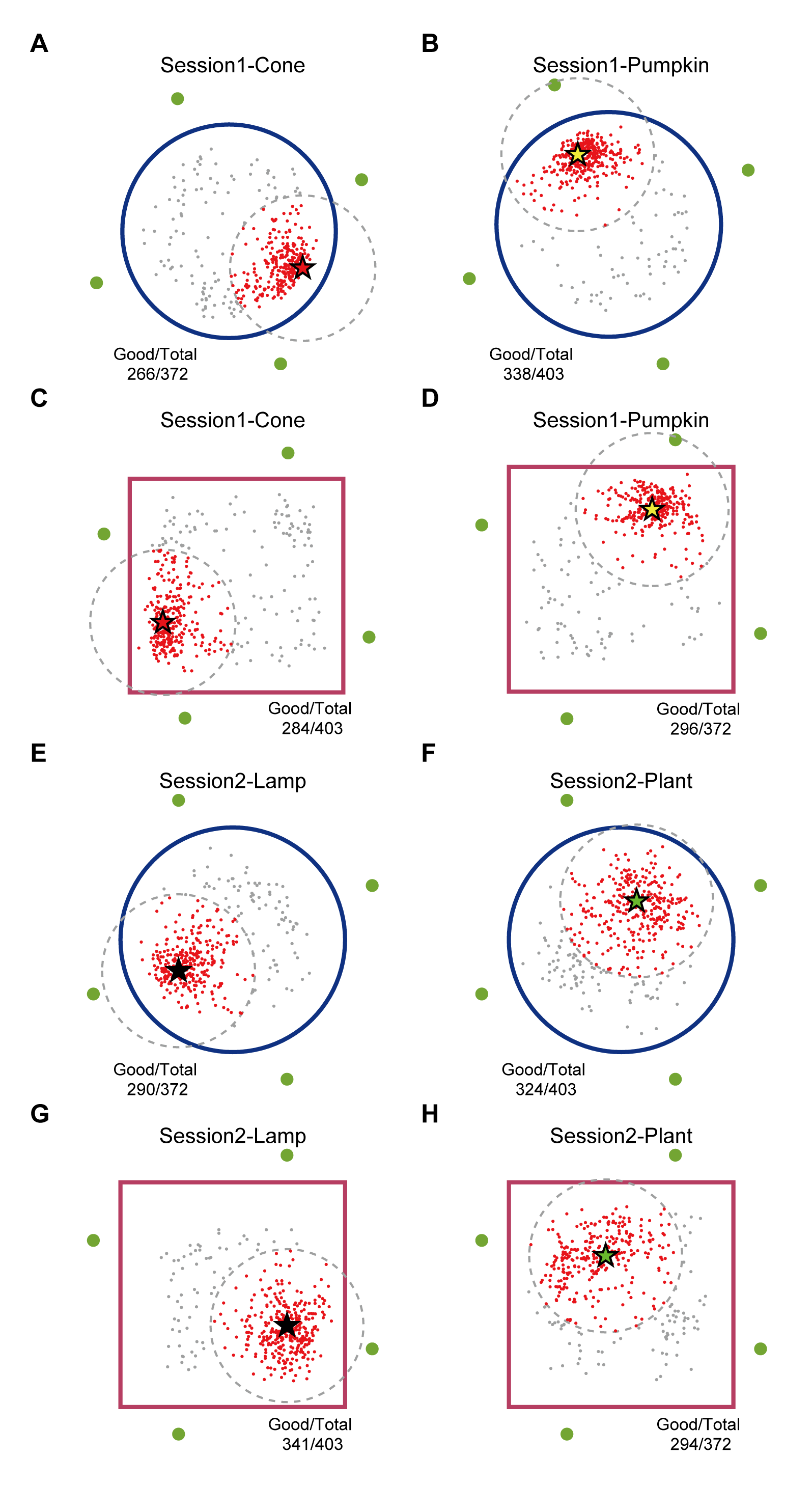

Supplement: S1 Fig — (A) Drop positions for the cone in the circle environment. (B) Drop positions for the pumpkin in the circle environment. (C) Drop positions for the cone in the square environment. (D) Drop positions for the pumpkin in the square environment. (E) Drop positions for the lamp in the circle environment. (F) Drop positions for the plant in the circle environment. (G) Drop positions for the lamp in the square environment. (H) Drop positions for the plant in the square environment. Blue circles in A, B, E, and F are the boundary of circle environment. Red squares in C, D, G, and H are the boundary of square environment. Solid green dots outside the boundaries indicate trees. Small red dots inside the boundaries are drop positions of good trials. Small gray dots inside the boundaries are drop positions of bad trials. Different color star represents different object. The red star represents the cone. The yellow star represents the pumpkin. The black star represents the lamp. The green star represents the plant. The dashed-line circles represent a radius of 9.87 vm centered on the current target object. Any trials placed the object within this area are deemed good trials. (TIF) [file pbio.3003398.s001.tif]

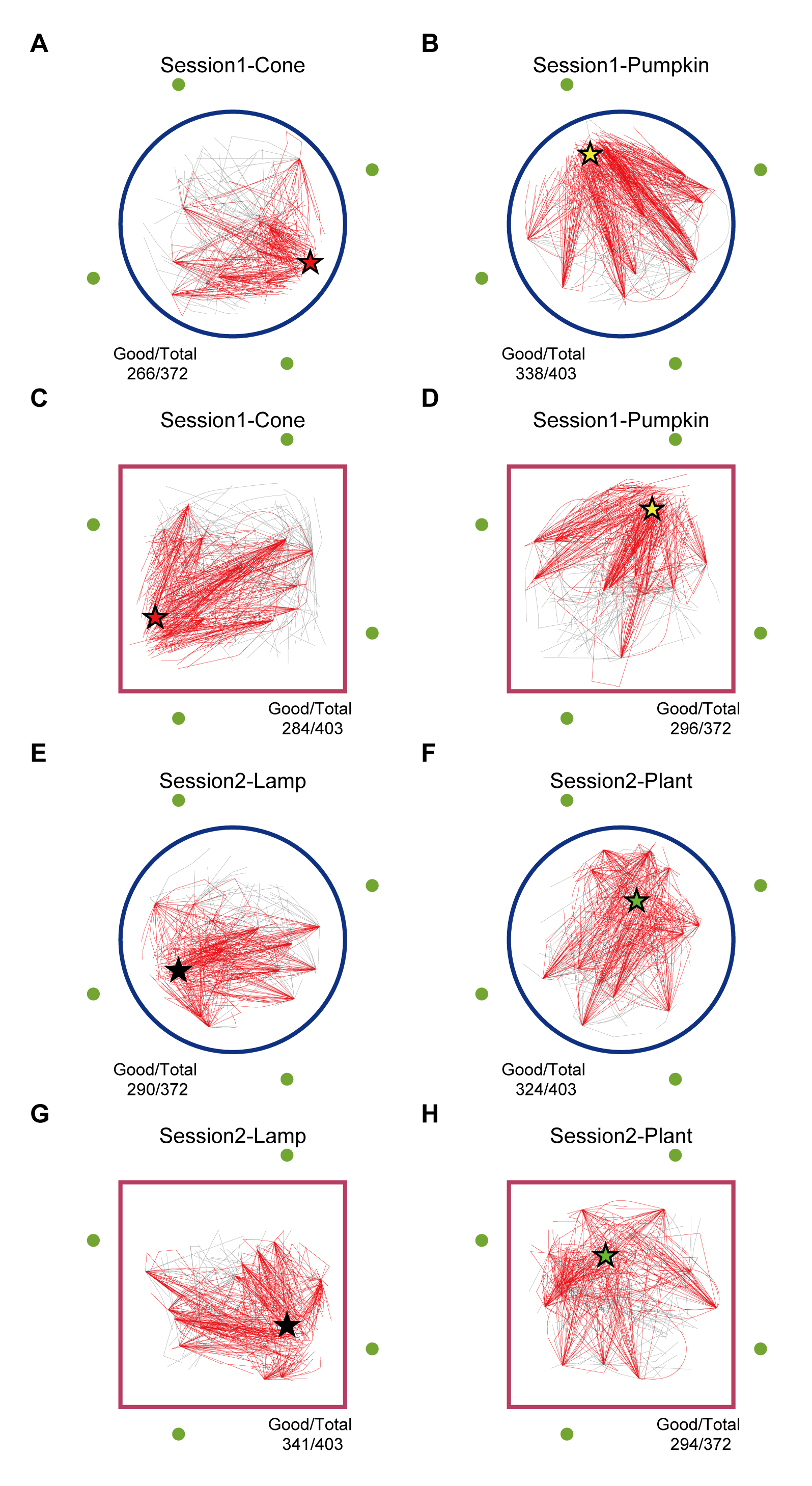

Supplement: S2 Fig — (A) Trajectories toward the cone in the circle environment. (B) Trajectories toward the pumpkin in the circle environment. (C) Trajectories toward the cone in the square environment. (D) Trajectories toward the pumpkin in the square environment. (E) Trajectories toward the lamp in the circle environment. (F) Trajectories toward the plant in the circle environment. (G) Trajectories toward the lamp in the square environment. (H) Trajectories toward the plant in the square environment. Red lines are trajectories toward the target location for good trials. Gray lines are trajectories toward the target location for bad trials. Notably, the participants move more straight toward the target location for good trials. (TIF) [file pbio.3003398.s002.tif]

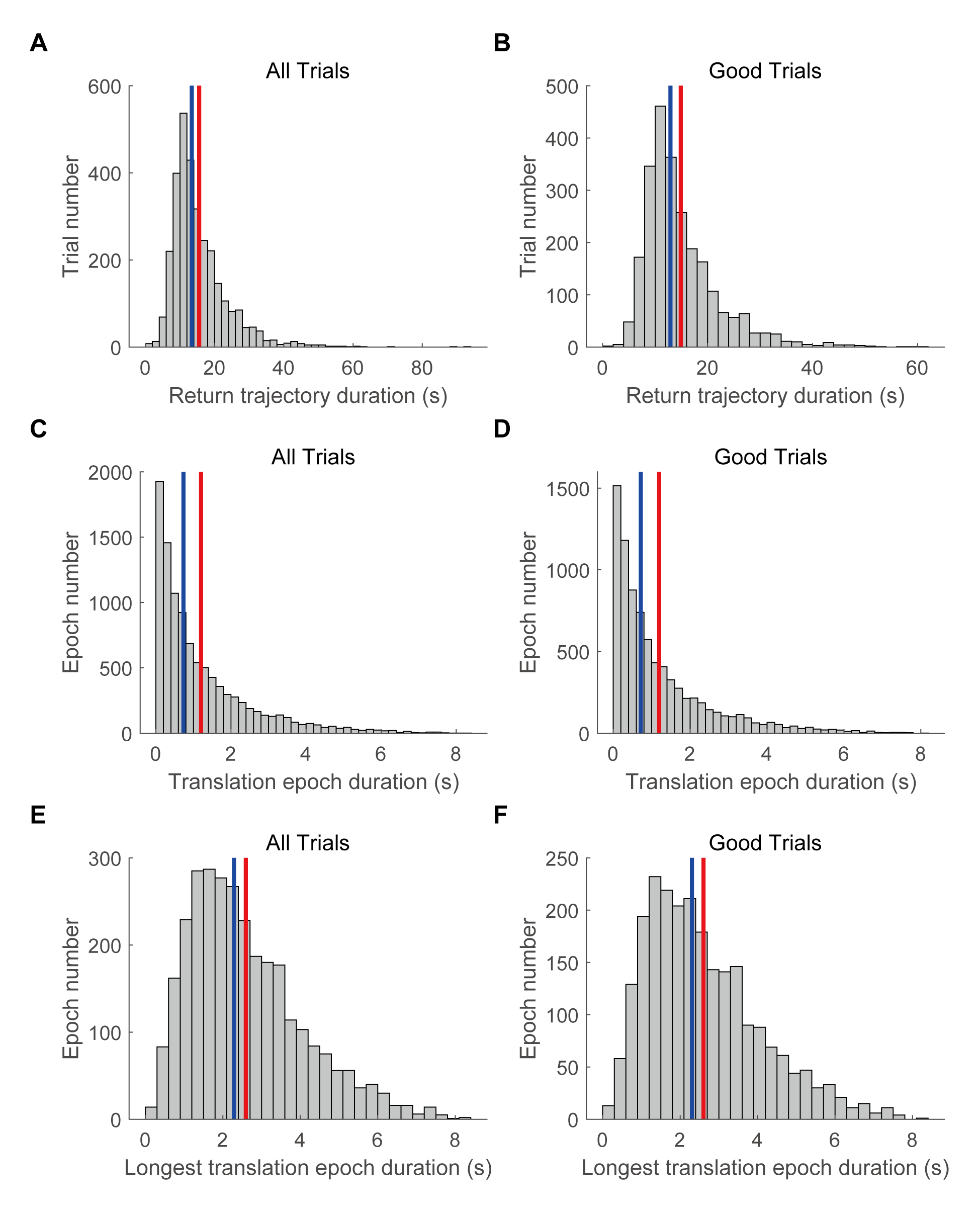

Supplement: S3 Fig — (A) The distribution of durations during which participants place objects back in all test trials. (B) The distribution of durations during which participants place objects back in good trials. (C) The distribution of durations for all translation movement epochs during which participants place objects back in all test trials. (D) The distribution of durations for all translation movement epochs during which participants place objects back in good trials. (E) The distribution of durations for the longest translation movement epoch in the return period of each test trial. (F) The distribution of durations for the longest translation movement epoch in the return period of each good test trial. In each panel, the red line represents the mean of the distribution, and the blue line represents the median of the distribution. (TIF) [file pbio.3003398.s003.tif]

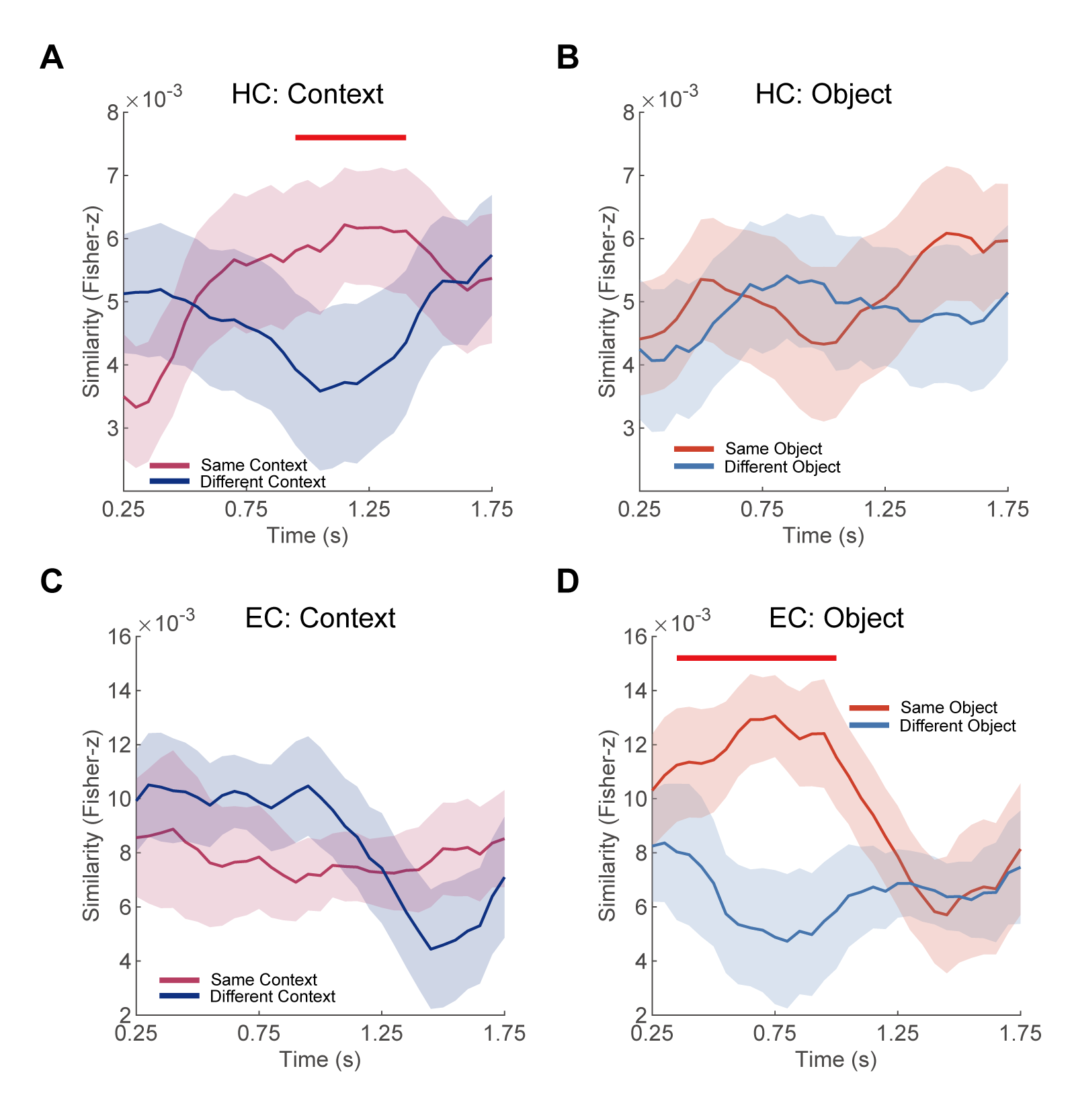

Supplement: S4 Fig — (A) For the translation epochs from all trials, the similarity of Same Context was significantly greater than the similarity of Different Context in HC. The horizontal red line marks the time window where these significant differences were observed. (B) For the translation epochs from all trials, there was no significant difference between the similarity of Same Object and the similarity of Different Object in HC. (C) For the translation epochs from all trials, there was no significant difference between the similarity of Same Context and the similarity of Different Context in EC. (D) For the translation epochs from all trials, the similarity of Same Object was significantly greater than the similarity of Different Object in EC. The horizontal red line indicated the time window where these significant differences were observed. The shaded areas represent SEM across participants. (TIF) [file pbio.3003398.s004.tif]

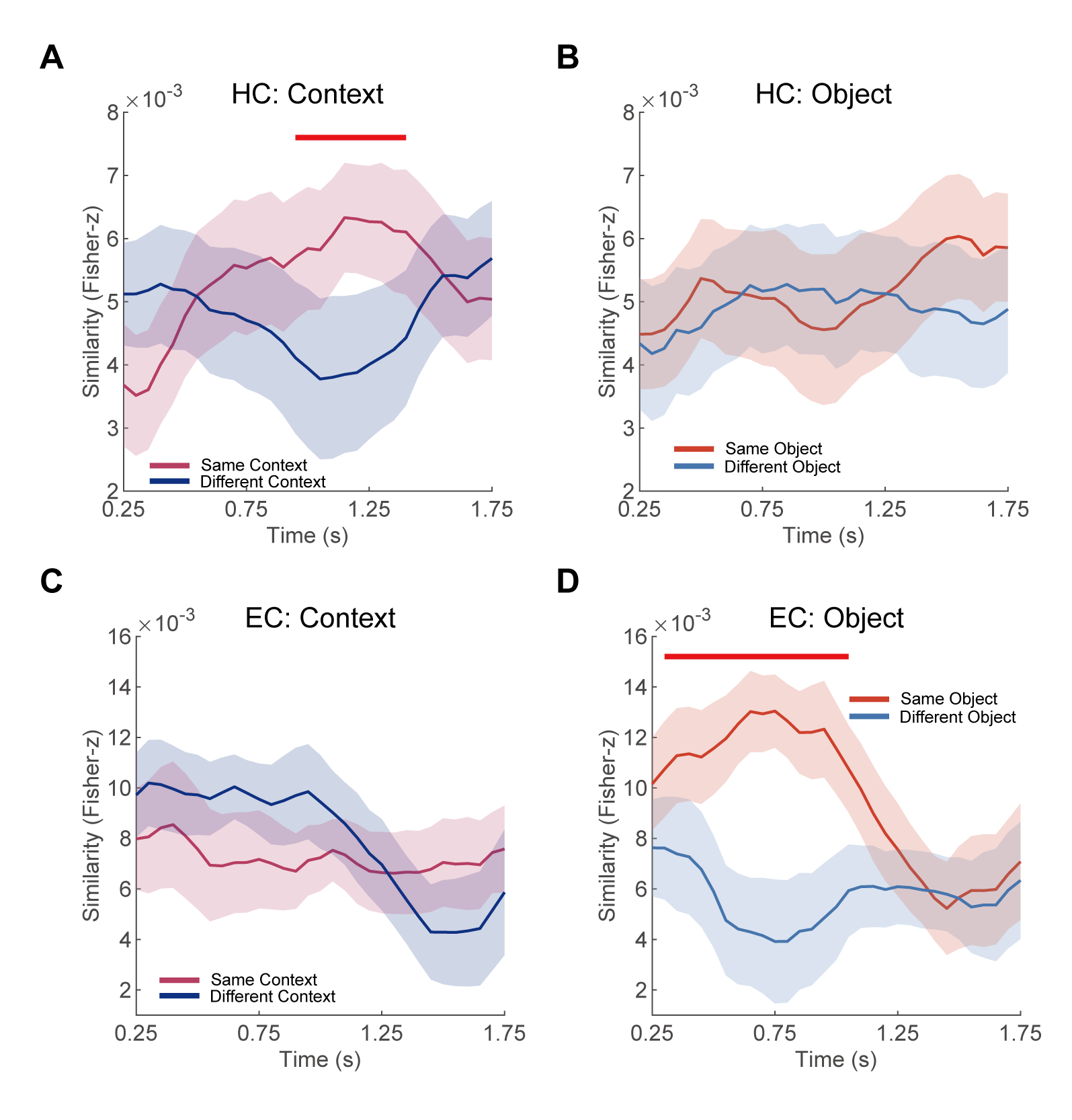

Supplement: S5 Fig — (A) For the translation epochs from all trials, the similarity of Same Context was significantly greater than the similarity of Different Context in HC. The horizontal red line marks the time window where these significant differences were observed. (B) For the translation epochs from all trials, there was no significant difference between the similarity of Same Object and the similarity of Different Object in HC. (C) For the translation epochs from all trials, there was no significant difference between the similarity of Same Context and the similarity of Different Context in EC. (D) For the translation epochs from all trials, the similarity of Same Object was significantly greater than the similarity of Different Object in EC. The horizontal red line indicated the time window where these significant differences were observed. The shaded areas represent SEM across participants. (TIF) [file pbio.3003398.s005.tif]

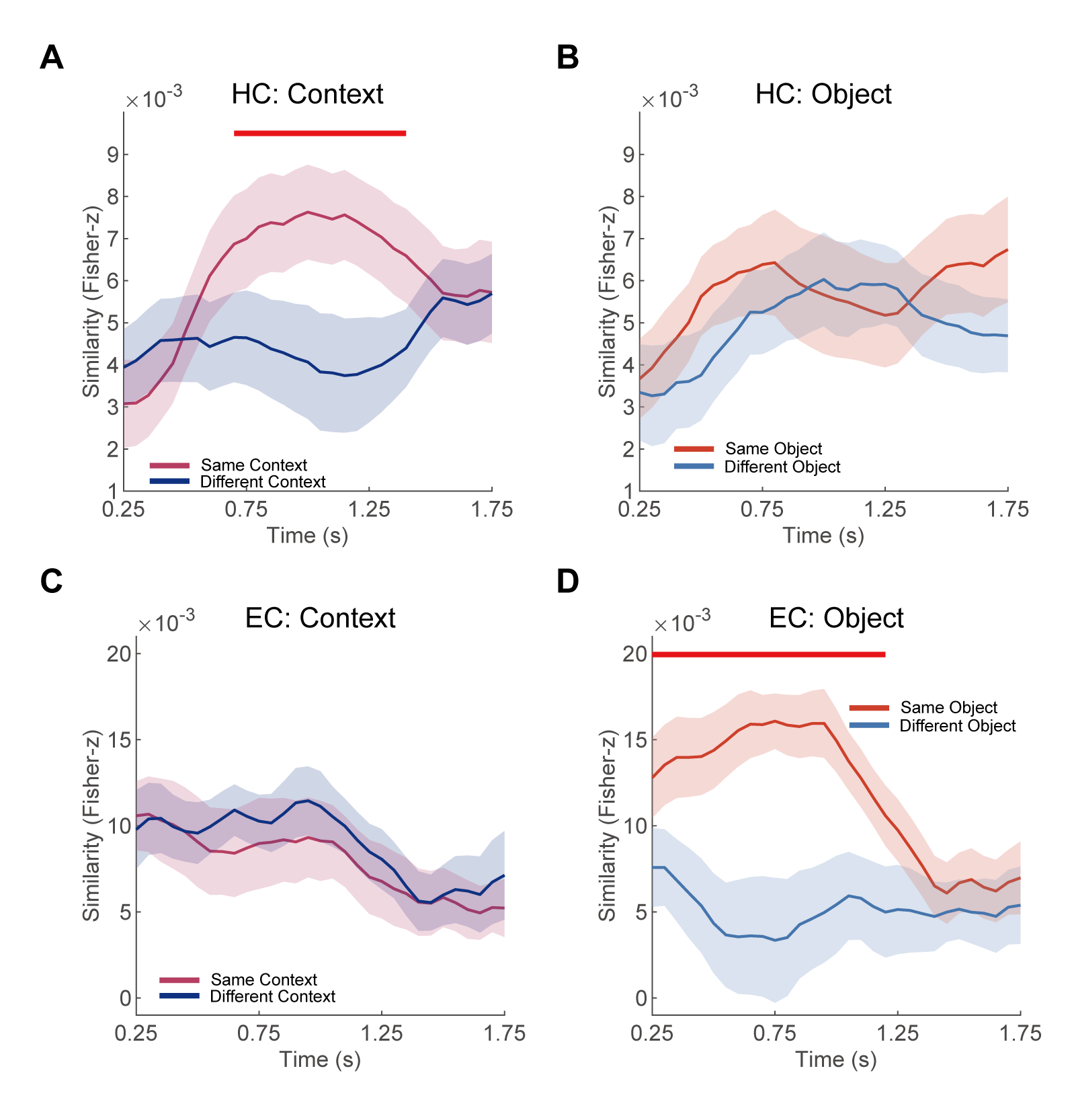

Supplement: S6 Fig — (A) For the translation epochs from good trials, the similarity of Same Context was significantly greater than the similarity of Different Context in HC. The horizontal red line marks the time window where these significant differences were observed. (B) For the translation epochs from good trials, there was no significant difference between the similarity of Same Object and the similarity of Different Object in HC. (C) For the translation epochs from good trials, there was no significant difference between the similarity of Same Context and the similarity of Different Context in EC. (D) For the translation epochs from good trials, the similarity of Same Object was significantly greater than the similarity of Different Object in EC. The horizontal red line indicated the time window where these significant differences were observed. The shaded areas represent SEM across participants. (TIF) [file pbio.3003398.s006.tif]

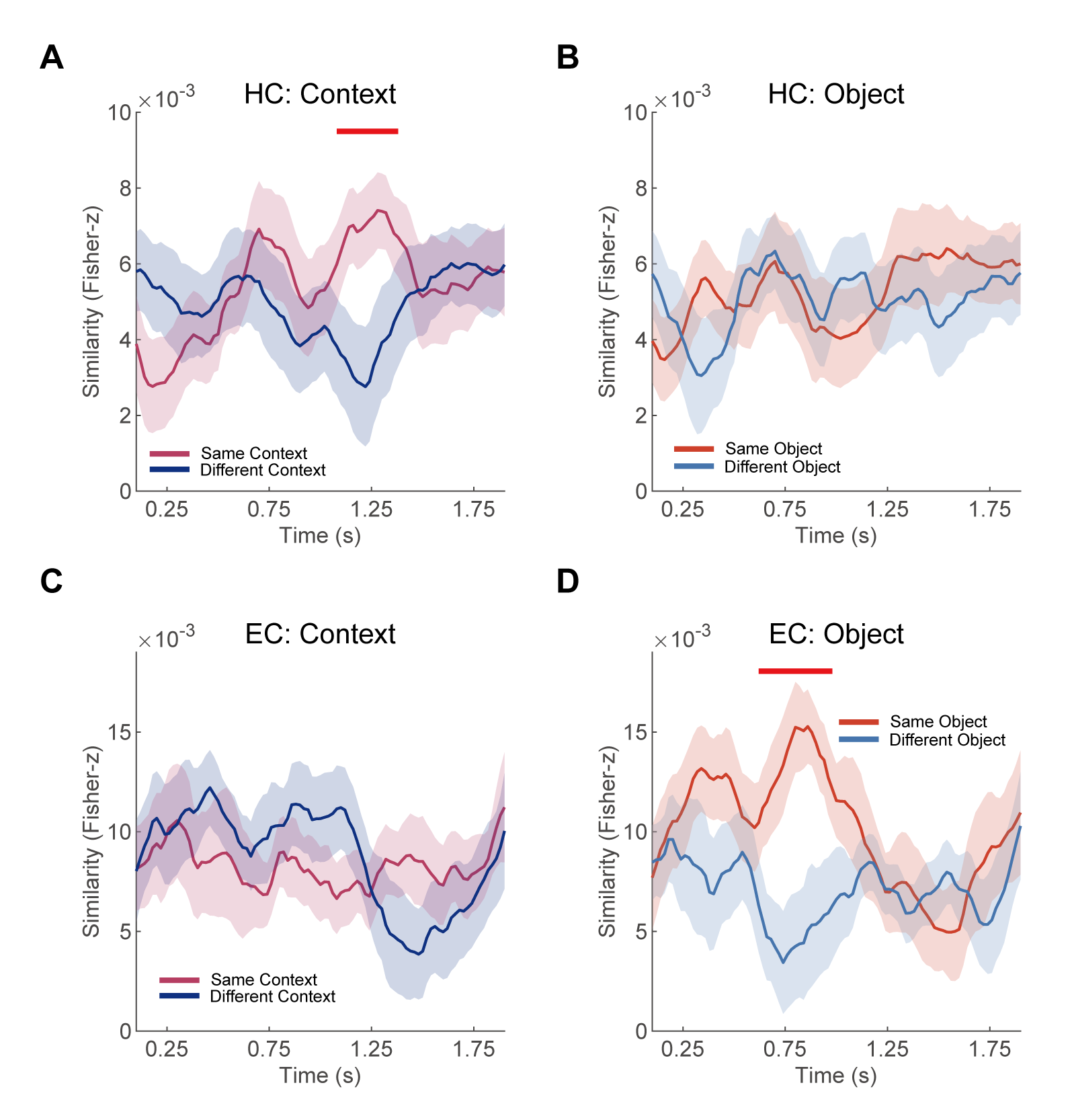

Supplement: S7 Fig — (A) For the translation epochs from all trials, the similarity of Same Context was significantly greater than the similarity of Different Context in HC. The horizontal red line marks the time window where these significant differences were observed. (B) For the translation epochs from all trials, there was no significant difference between the similarity of Same Object and the similarity of Different Object in HC. (C) For the translation epochs from all trials, there was no significant difference between the similarity of Same Context and the similarity of Different Context in EC. (D) For the translation epochs from all trials, the similarity of Same Object was significantly greater than the similarity of Different Object in EC. The horizontal red line indicated the time window where these significant differences were observed. The shaded areas represent SEM across participants. (TIF) [file pbio.3003398.s007.tif]

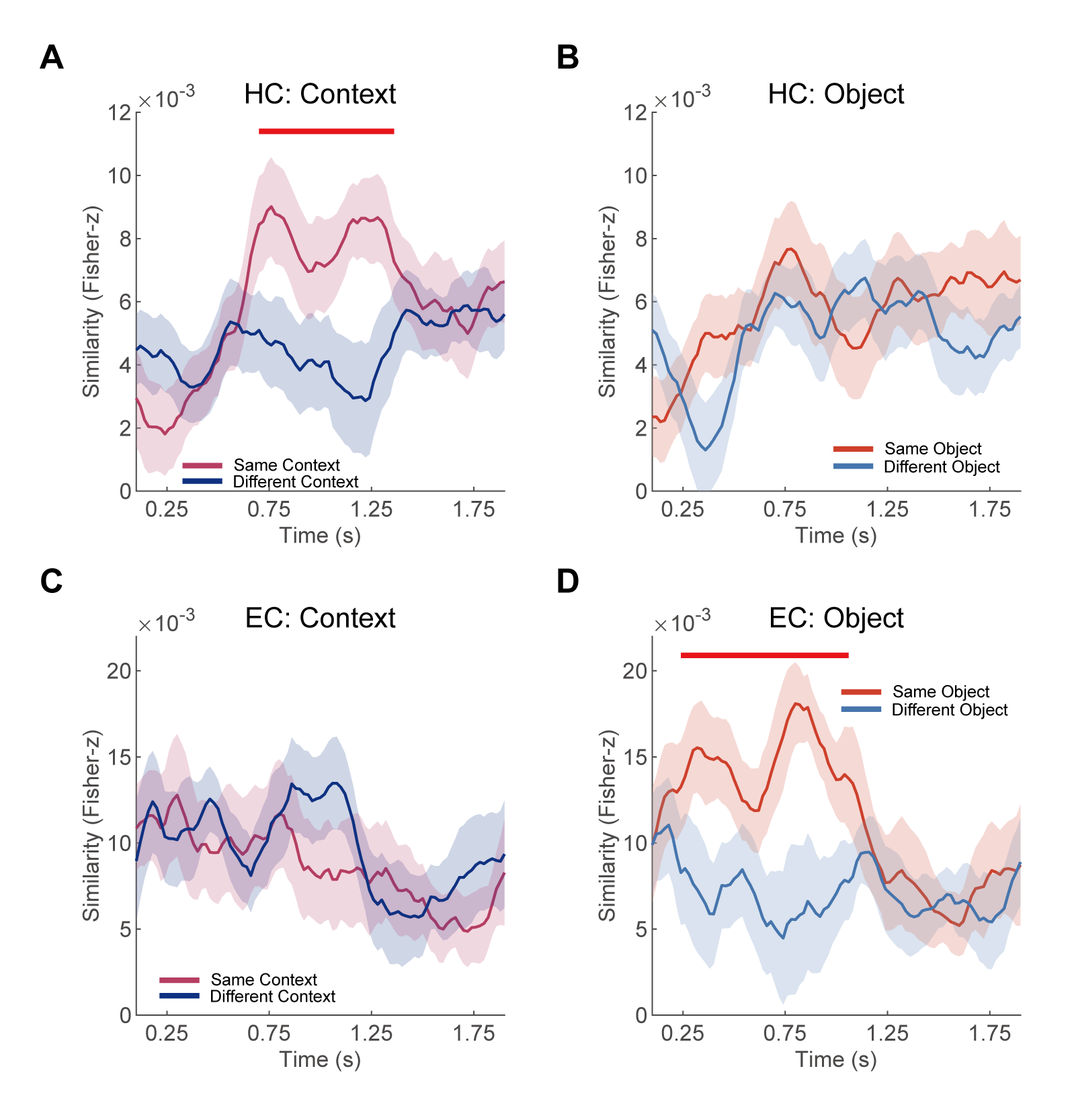

Supplement: S8 Fig — (A) For the translation epochs from good trials, the similarity of Same Context was significantly greater than the similarity of Different Context in HC. The horizontal red line marks the time window where these significant differences were observed. (B) For the translation epochs from good trials, there was no significant difference between the similarity of Same Object and the similarity of Different Object in HC. (C) For the translation epochs from good trials, there was no significant difference between the similarity of Same Context and the similarity of Different Context in EC. (D) For the translation epochs from good trials, the similarity of Same Object was significantly greater than the similarity of Different Object in EC. The horizontal red line indicated the time window where these significant differences were observed. The shaded areas represent SEM across participants. (TIF) [file pbio.3003398.s008.tif]

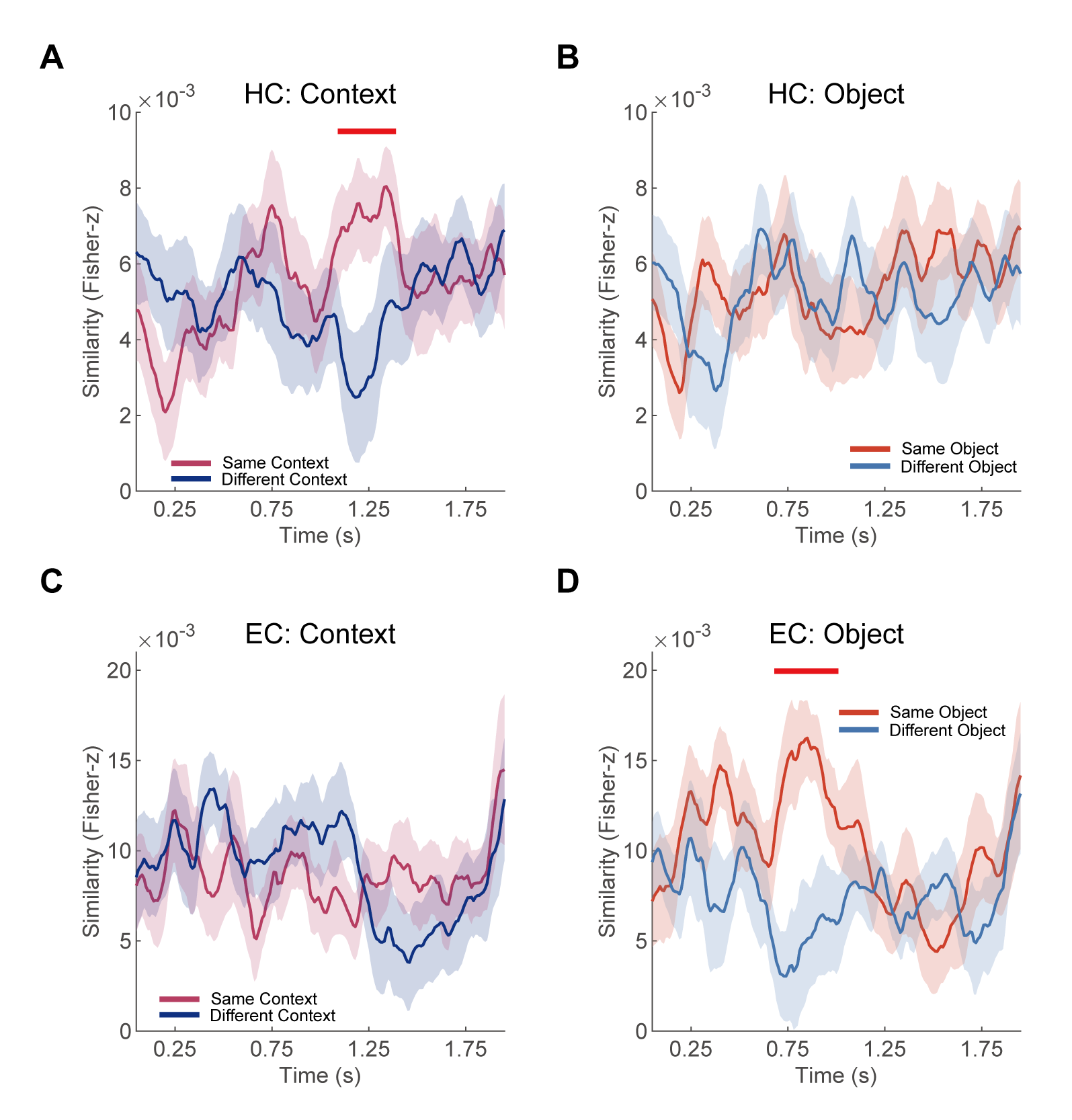

Supplement: S9 Fig — (A) For the translation epochs from all trials, the similarity of Same Context was significantly greater than the similarity of Different Context in HC. The horizontal red line marks the time window where these significant differences were observed. (B) For the translation epochs from all trials, there was no significant difference between the similarity of Same Object and the similarity of Different Object in HC. (C) For the translation epochs from all trials, there was no significant difference between the similarity of Same Context and the similarity of Different Context in EC. (D) For the translation epochs from all trials, the similarity of Same Object was significantly greater than the similarity of Different Object in EC. The horizontal red line indicated the time window where these significant differences were observed. The shaded areas represent SEM across participants. (TIF) [file pbio.3003398.s009.tif]

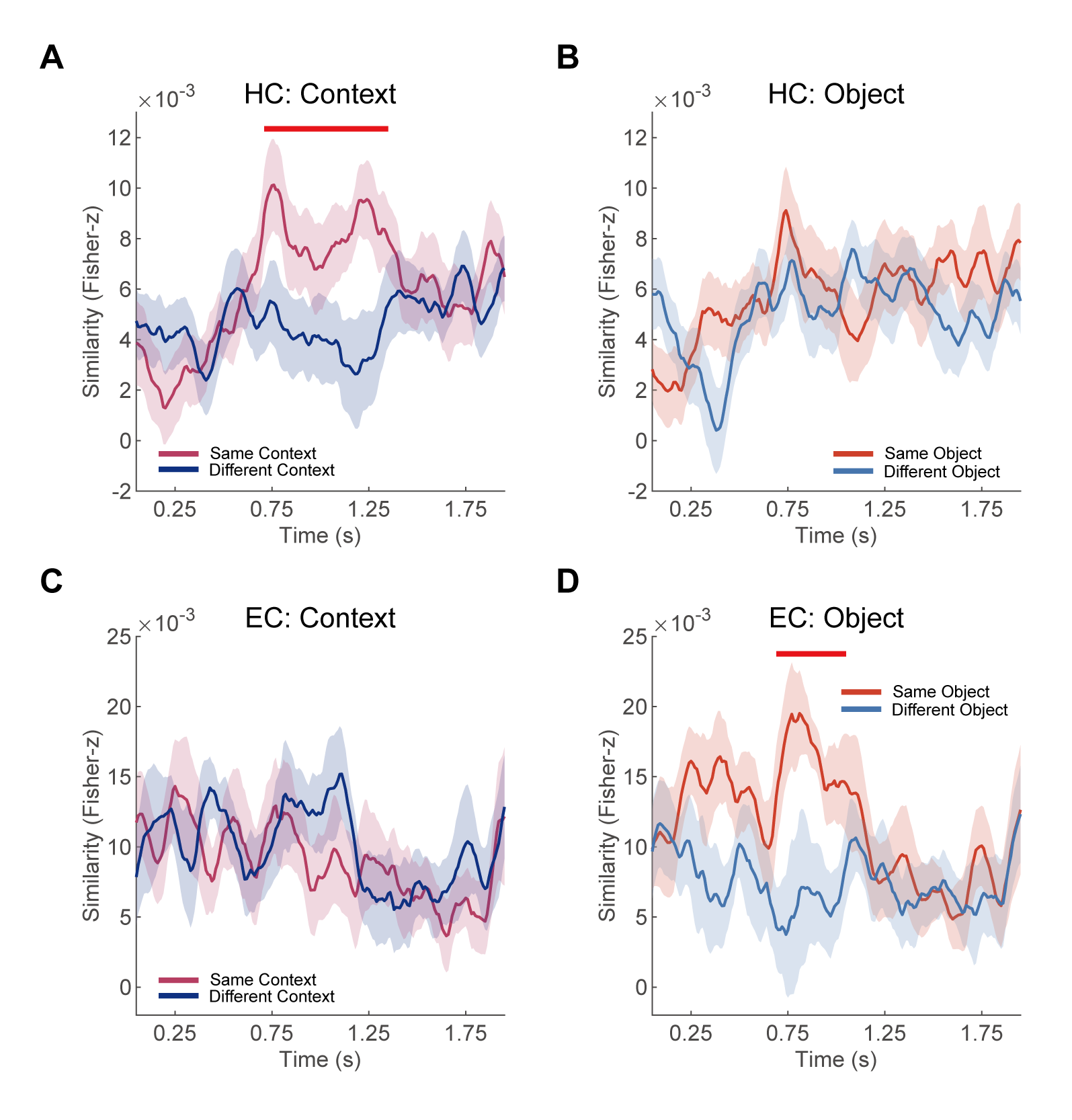

Supplement: S10 Fig — (A) For the translation epochs from good trials, the similarity of Same Context was significantly greater than the similarity of Different Context in HC. The horizontal red line marks the time window where these significant differences were observed. (B) For the translation epochs from good trials, there was no significant difference between the similarity of Same Object and the similarity of Different Object in HC. (C) For the translation epochs from good trials, there was no significant difference between the similarity of Same Context and the similarity of Different Context in EC. (D) For the translation epochs from good trials, the similarity of Same Object was significantly greater than the similarity of Different Object in EC. The horizontal red line indicated the time window where these significant differences were observed. The shaded areas represent SEM across participants. (TIF) [file pbio.3003398.s010.tif]

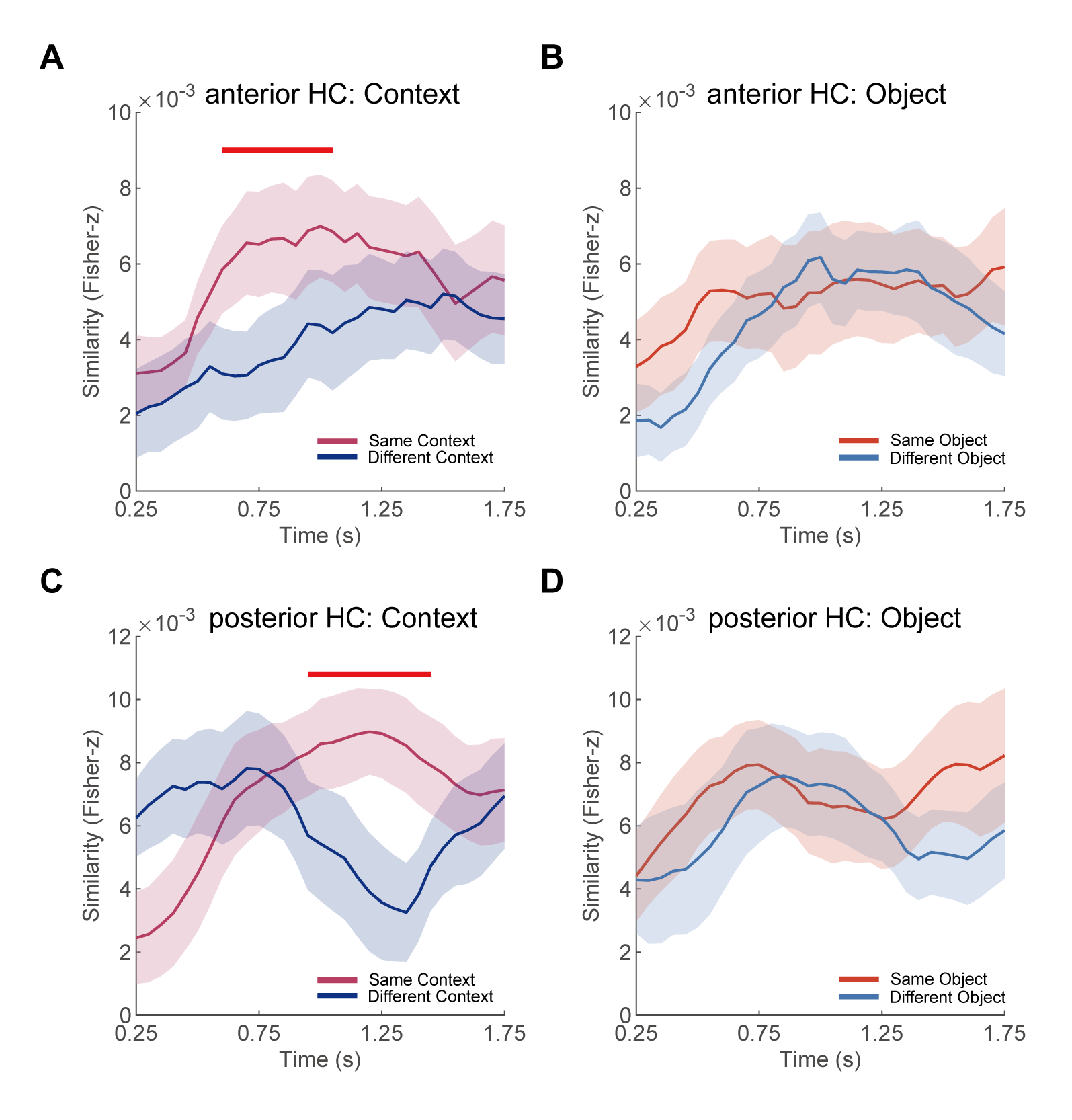

Supplement: S11 Fig — (A) For the activity in the anterior HC, the similarity of Same Context was significantly greater than the Different Context. The horizontal red line indicated the time window where these significant differences were observed. (B) For the activity in the anterior HC, there was no significant similarity difference between Same Object and Different Object. (C) For the activity in the posterior HC, the similarity of Same Context was significantly greater than Different Context. The horizontal red line marks the time window where these significant differences were observed. (D) For the activity in the posterior HC, there was no significant similarity difference between Same Object and Different Object. The shaded areas represent SEM across participants. (TIF) [file pbio.3003398.s011.tif]

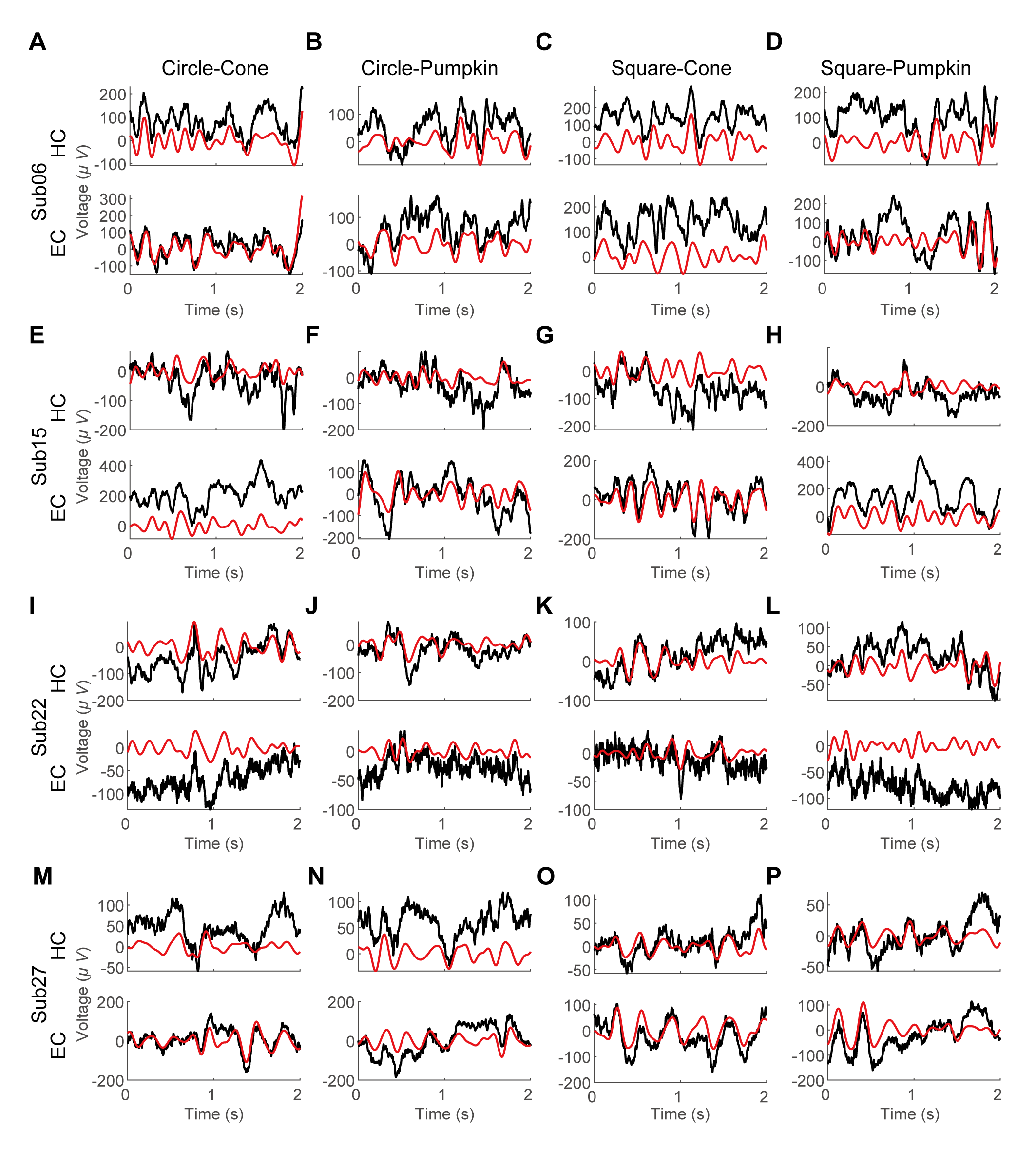

Supplement: S12 Fig — (A–D) Raw signals (black lines) and filter low-frequency (2–8 Hz) signals (red lines) of four translation epochs from a HC electrode and one EC electrode of Sub06. (E–H) Raw signals (black lines) and filter low-frequency (2–8 Hz) signals (red lines) of four translation epochs from a HC electrode and one EC electrode of Sub15. (I–L) Raw signals (black lines) and filter low-frequency (2–8 Hz) signals (red lines) of four translation epochs from a HC electrode and one EC electrode of Sub22. (M–P) Raw signals (black lines) and filter low-frequency (2–8 Hz) signals (red lines) of four translation epochs from a HC electrode and one EC electrode of Sub27. (TIF) [file pbio.3003398.s012.tif]

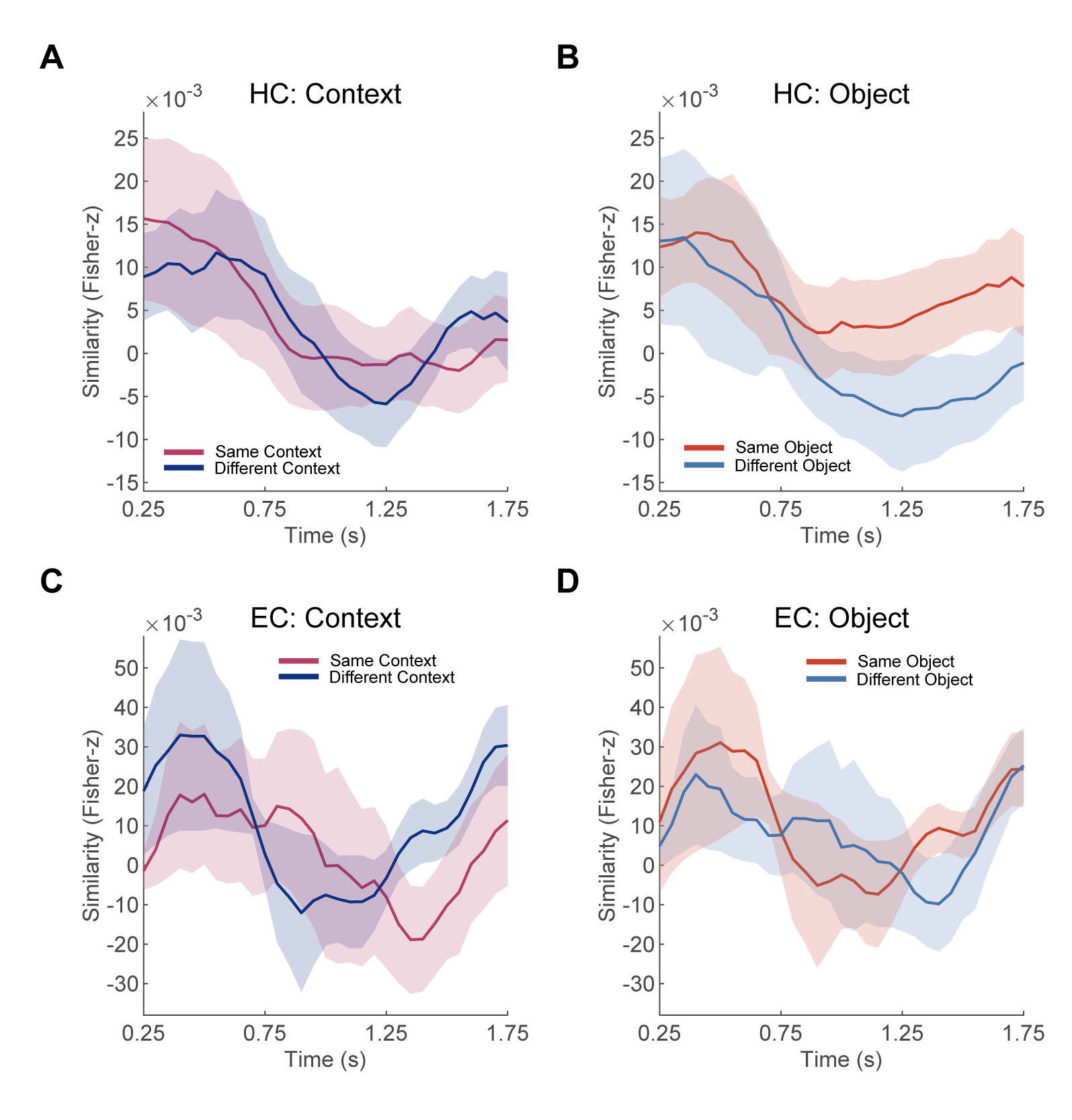

Supplement: S13 Fig — For the translation epochs from bad trials, neither HC nor EC showed significant differences in neural representational similarity between Same Context versus Different Context conditions (A and C) or between Same Object versus Different Object conditions (B and D). The shaded areas represent SEM across participants. (TIF) [file pbio.3003398.s013.tif]

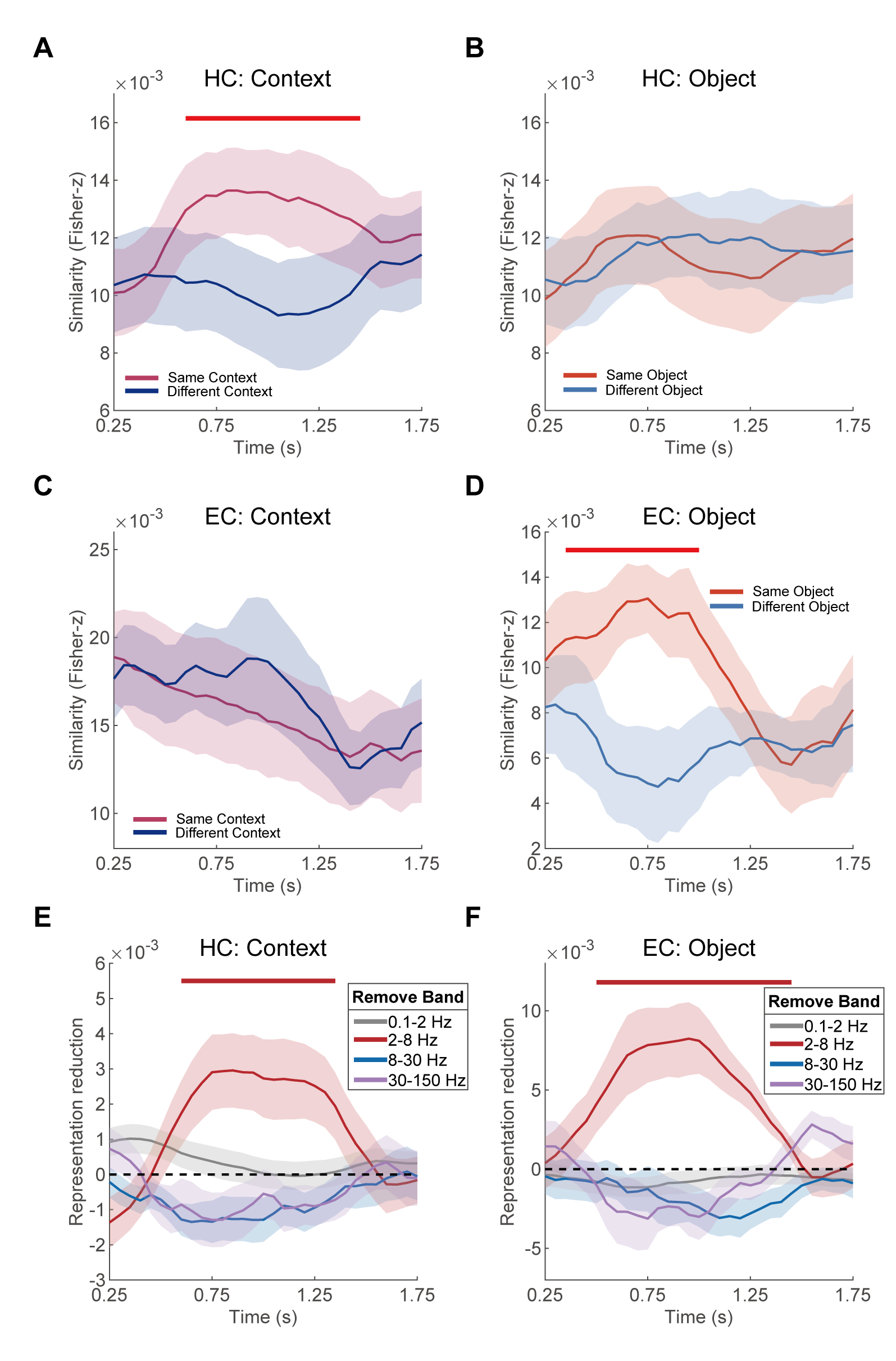

Supplement: S14 Fig — (A) For the translation epochs from good trials, the similarity of Same Context was significantly greater than the similarity of Different Context in HC. The horizontal red line marks the time window where these significant differences were observed. (B) For the translation epochs from good trials, there was no significant difference between the similarity of Same Object and the similarity of Different Object in HC. (C) For the translation epochs from good trials, there was no significant difference between the similarity of Same Context and the similarity of Different Context in EC. (D) For the translation epochs from good trials, the similarity of Same Object was significantly greater than the similarity of Different Object in EC. The horizontal red line indicated the time window where these significant differences were observed. (E) In broader-frequency-band RSA results, removal of 2–8 Hz activity significantly reduced HC context representation. The horizontal red line indicated the time window that was significantly greater than zero. However, removing activity in the 0.1–2 Hz, 8–30 Hz, or 30–150 Hz bands did not significantly reduce HC context representation. (F) In broader-frequency-band RSA results, removal of 2–8 Hz activity significantly reduced EC object representation. The horizontal red line indicated the time window that was significantly greater than zero. While there was no significant decrease of EC object representation after excluding 0.1–2 Hz, 8–30 Hz, or 30–150 Hz activity. The shaded areas represent SEM across participants. (TIF) [file pbio.3003398.s014.tif]

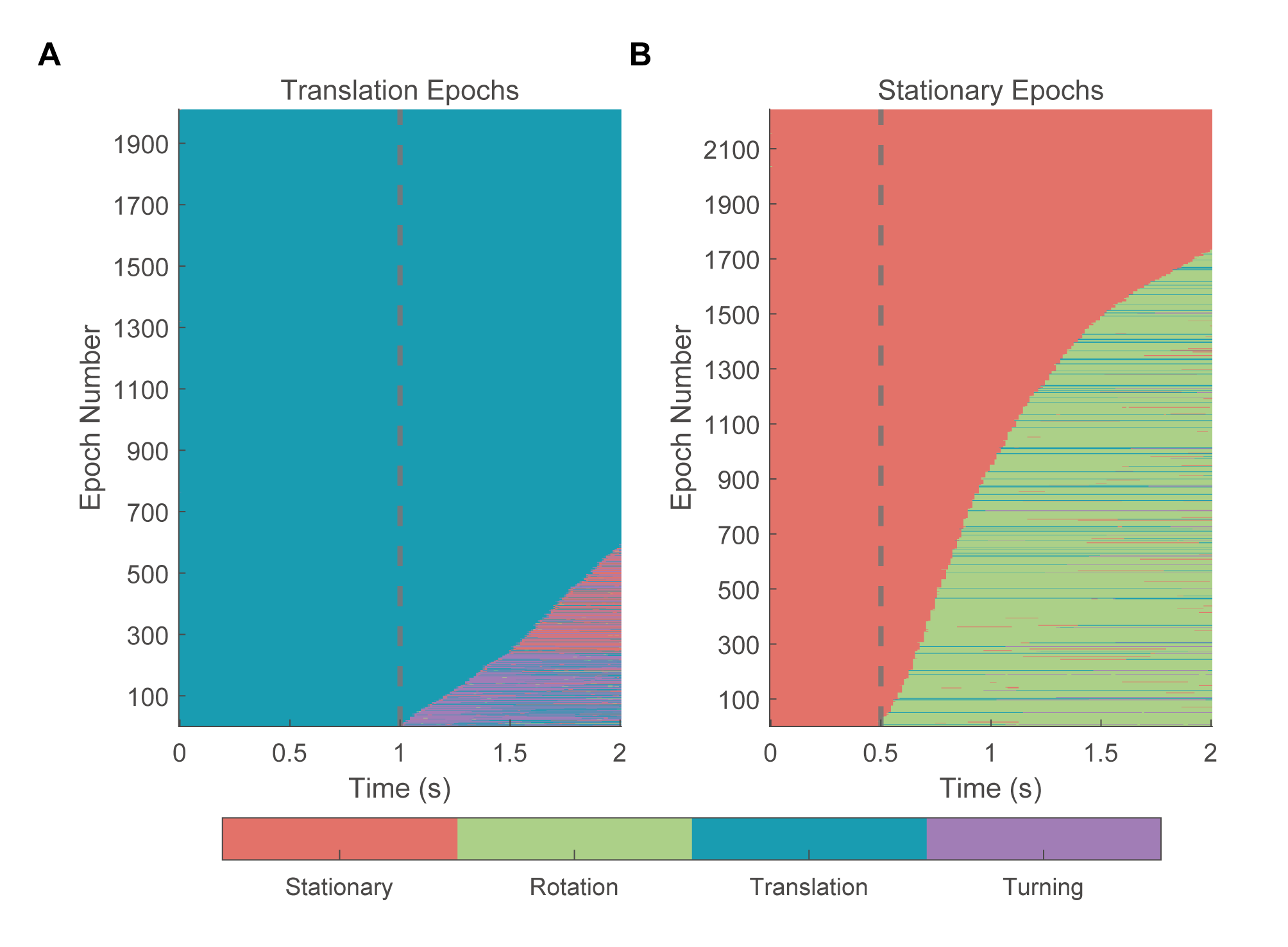

Supplement: S15 Fig — Stationary refers to states with zero linear velocity and zero angular velocity. Rotation is defined by zero linear velocity and non-zero angular velocity. Translation refers to states with non-zero linear velocity and zero angular velocity. Turing motion is defined by non-zero linear velocity and non-zero angular velocity. (TIF) [file pbio.3003398.s015.tif]

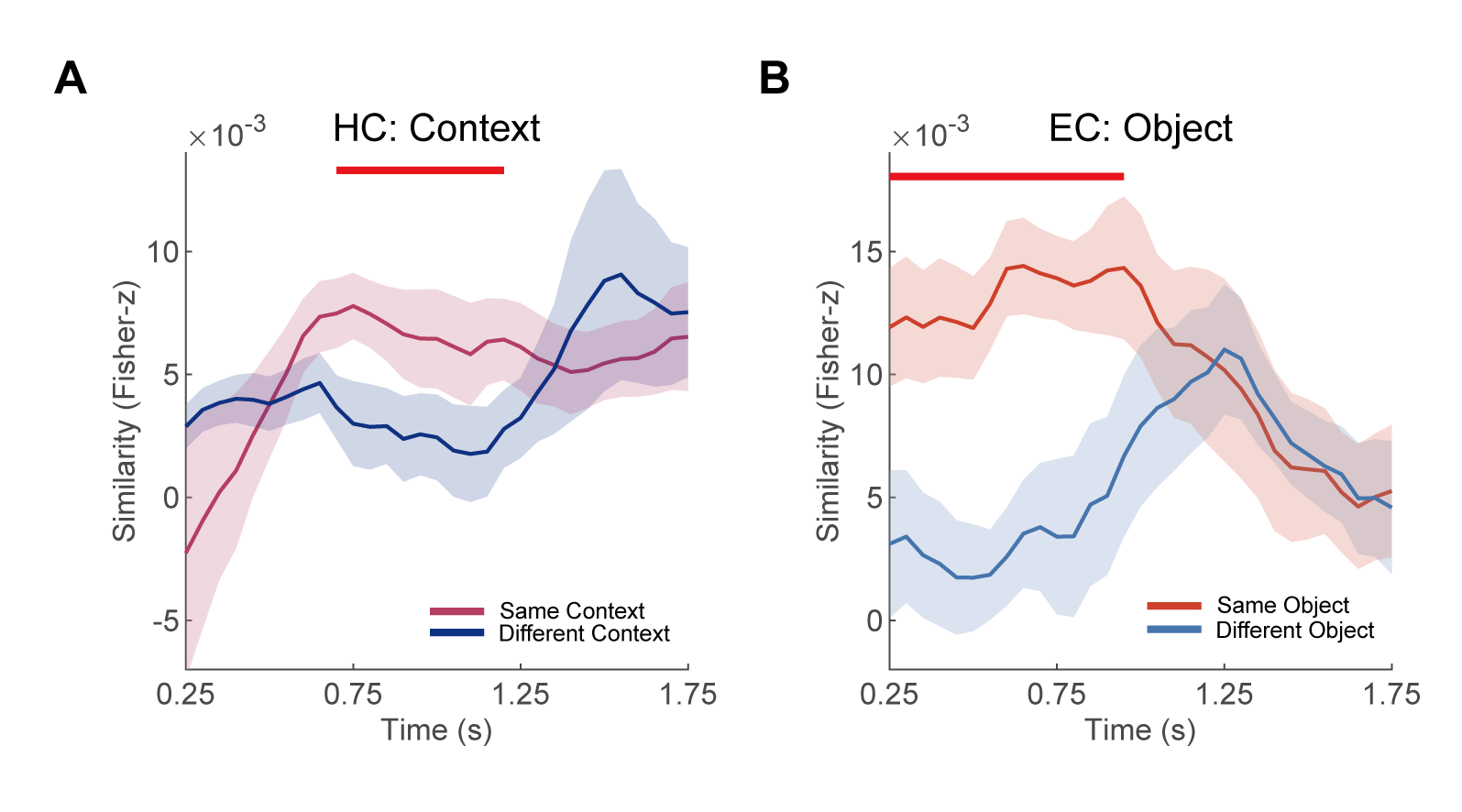

Supplement: S16 Fig — (A) For the translation epochs from good trials, the similarity of Same Context was significantly greater than the similarity of Different Context in HC. The horizontal red line marks the time window where these significant differences were observed. (B) For the translation epochs from good trials, the similarity of Same Object was significantly greater than the similarity of Different Object in EC. The horizontal red line indicated the time window where these significant differences were observed. The shaded areas represent SEM across participants. (TIF) [file pbio.3003398.s016.tif]

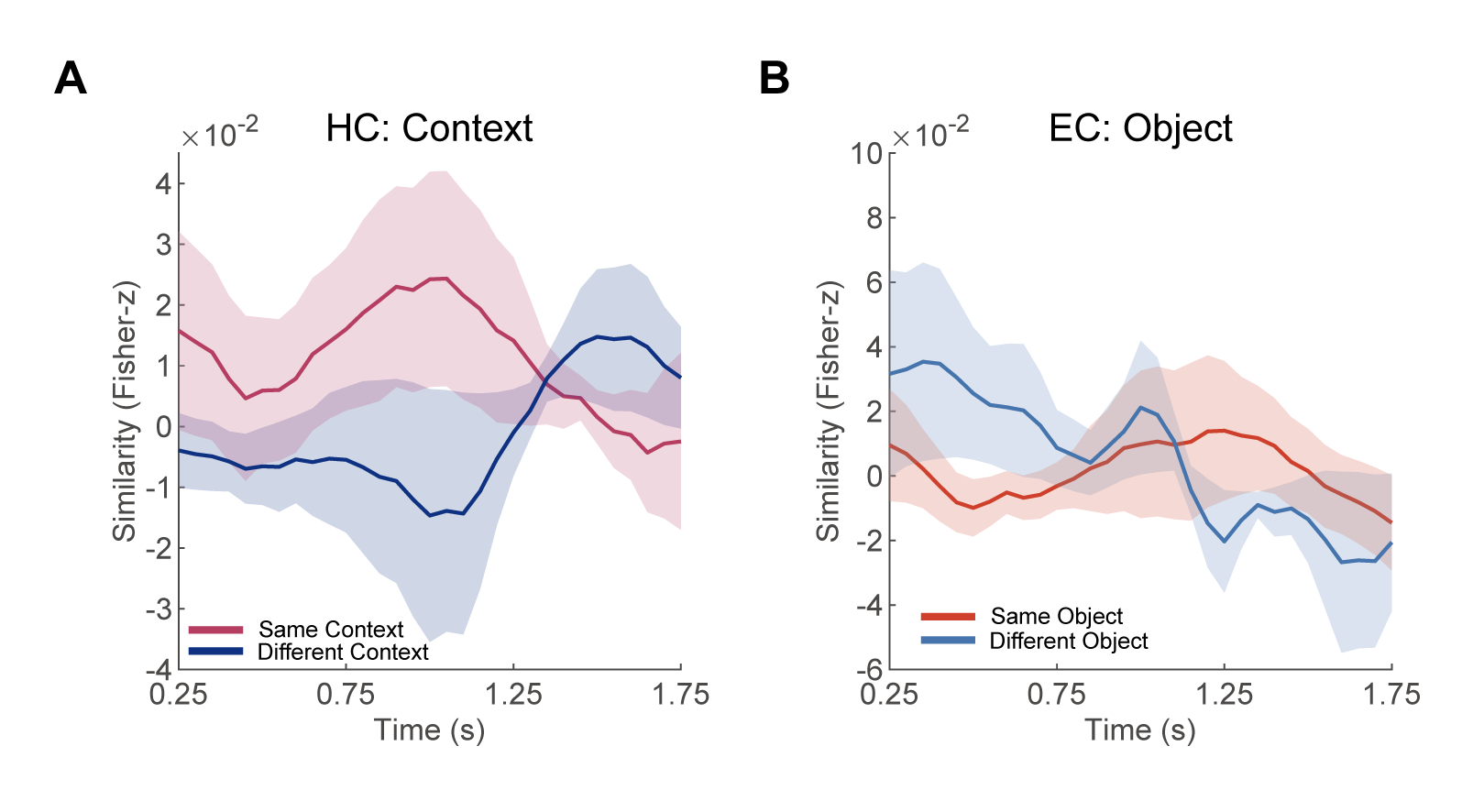

Supplement: S17 Fig — (A) For the stationary epochs from good trials, there was no significant difference between the similarity of Same Context and the similarity of Different Context in HC. (B) For the stationary epochs from good trials, there was no significant difference between the similarity of Same Object and the similarity of Different Object in EC. The shaded areas represent SEM across participants. (TIF) [file pbio.3003398.s017.tif]

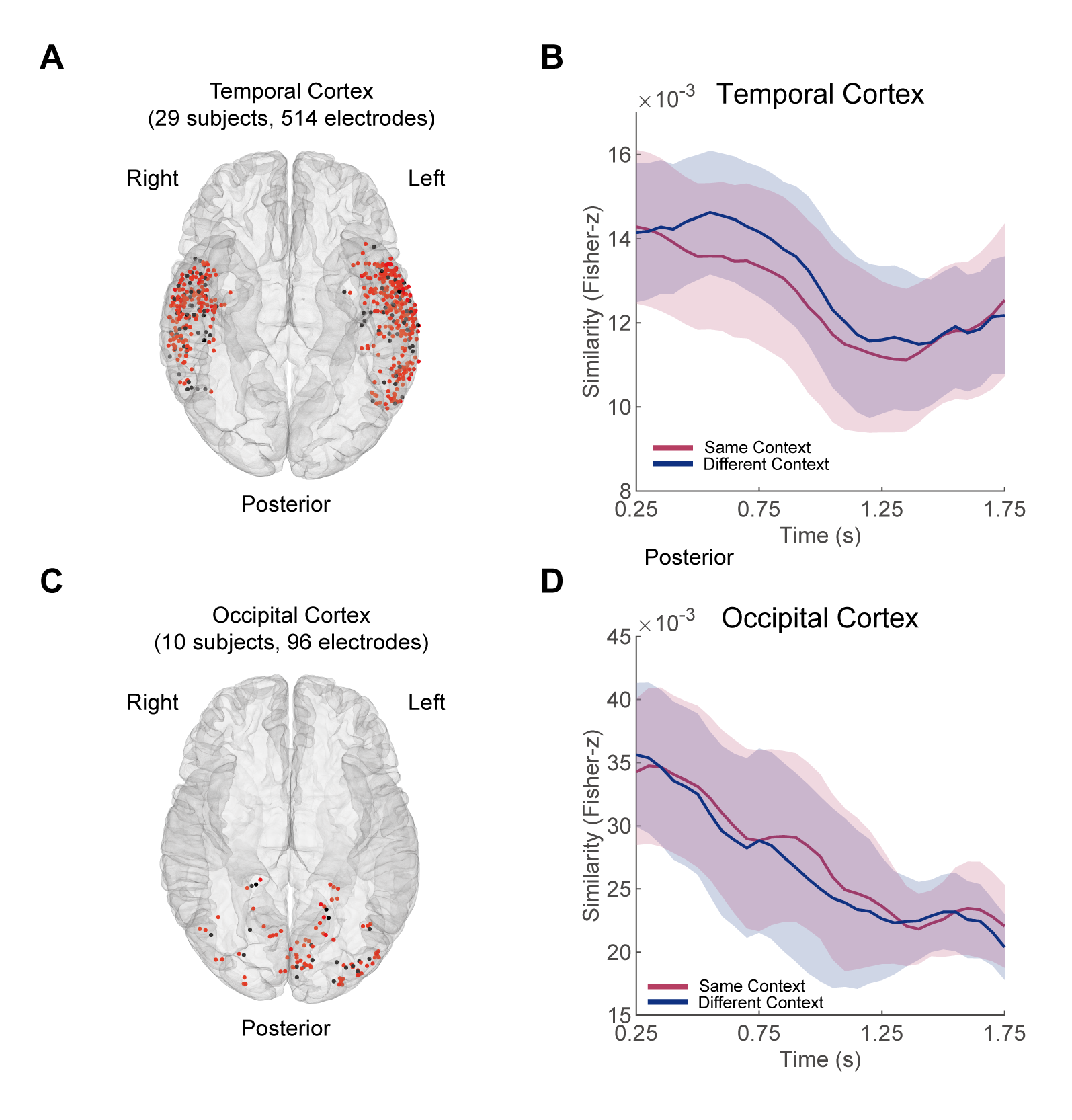

Supplement: S18 Fig — (A) The positions of temporal electrodes projected onto the MNI152 template from all 29 participants. The red dots are task-selective electrodes (N = 407). The black dots are non-task-selective electrodes (N = 107). (B) For the translation epochs from good trials, there was no significant difference between the similarity of Same Context and the similarity of Different Context in temporal cortex. (C) The positions of occipital electrodes projected onto the MNI152 template from all 10 participants. The red dots are task-selective electrodes (N = 76). The black dots are non-task-selective electrodes (N = 30). (D) For the translation epochs from good trials, there was no significant difference between the similarity of Same Context and the similarity of Different Context in occipital cortex. The shaded areas represent SEM across participants in panels B and D. (TIF) [file pbio.3003398.s018.tif]

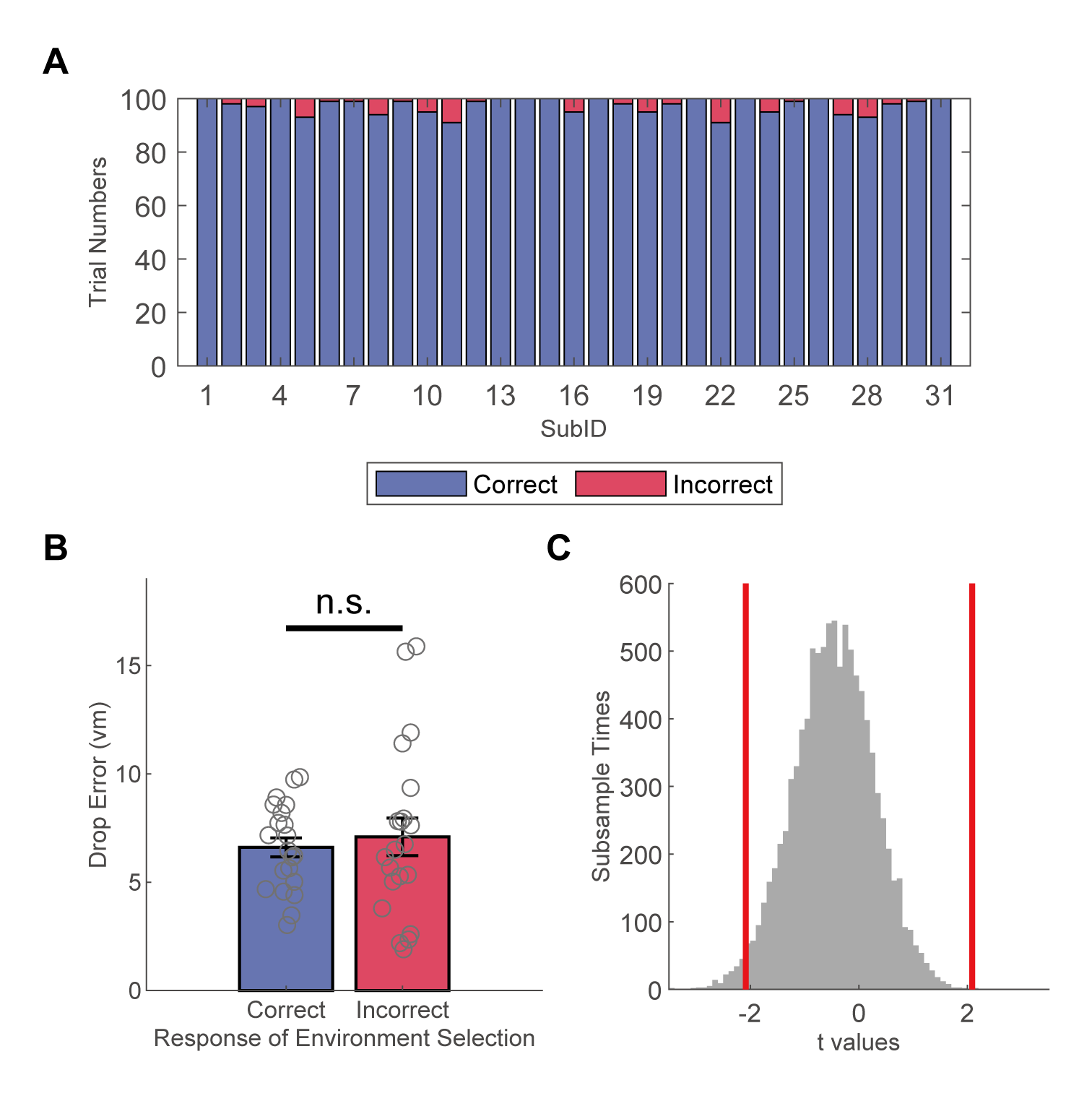

Supplement: S19 Fig — (A) Number of correct and incorrect context-selection trials for each participant. (B) Among participants with context-selection errors (N = 21), no significant difference was observed in mean drop error between correct and incorrect context-selection trials. N.s. denotes “non-significant”. Hollow dots are individual subjects, and error bars indicate SEM across participants. (C) Given the unequal number of correct and incorrect context-selection trials, we randomly selected a matched number of correct trials per participant (equal to their error trials), averaged their drop errors, and performed participant-level paired t-tests. This procedure was repeated 10,000 times, with approximately 196 iterations yielding t-values exceeding the critical threshold (two-tailed paired t test, df = 20, α = 0.05, tcritical = ±2.086, the red vertical lines, psubsample = 1–196/10000 = 0.9804). (TIF) [file pbio.3003398.s019.tif]

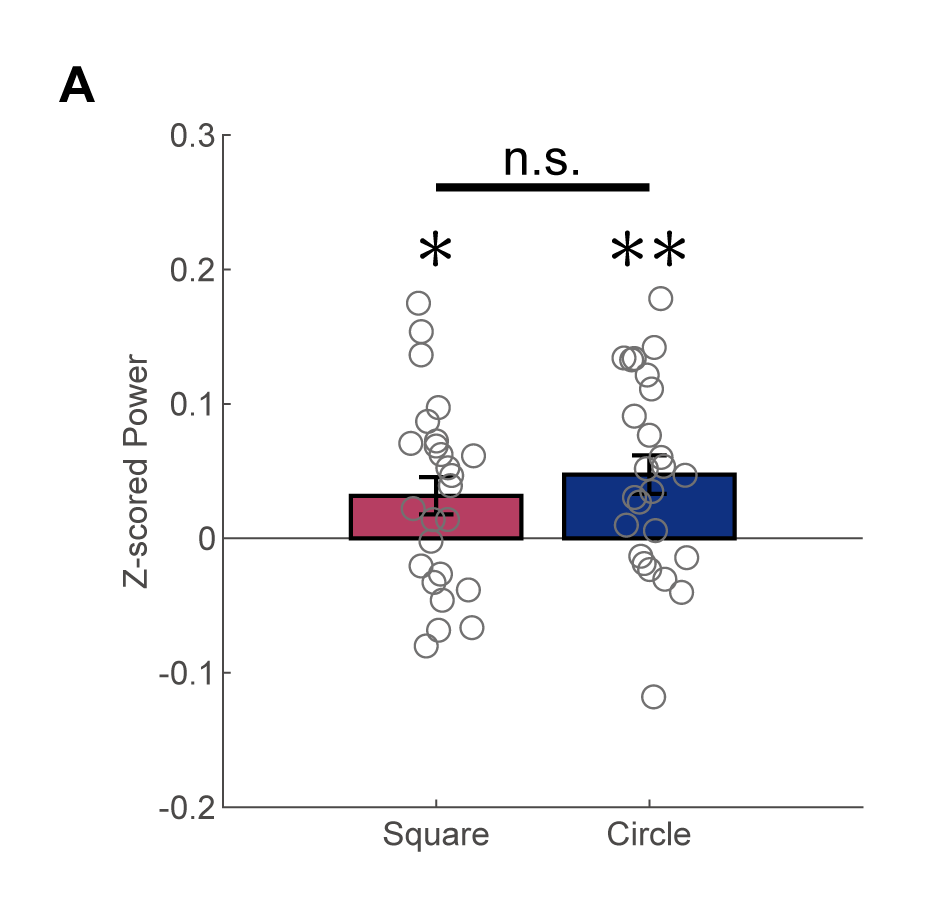

Supplement: S20 Fig — (A) Z-scored hippocampal theta power (2−8 Hz,) was significantly greater than zero during translation epochs from good trials in both the square (t24 = 2.279, p = 0.016) and circle (t24 = 3.309, p = 0.001) environments; furthermore, no significant difference emerged between environmental conditions (t24 = −0.911, p = 0.371). Hollow dots are individual subjects, and error bars indicate SEM across participants. N.s. denotes “non-significant”. * p < 0.05, ** p < 0.01. (TIF) [file pbio.3003398.s020.tif]

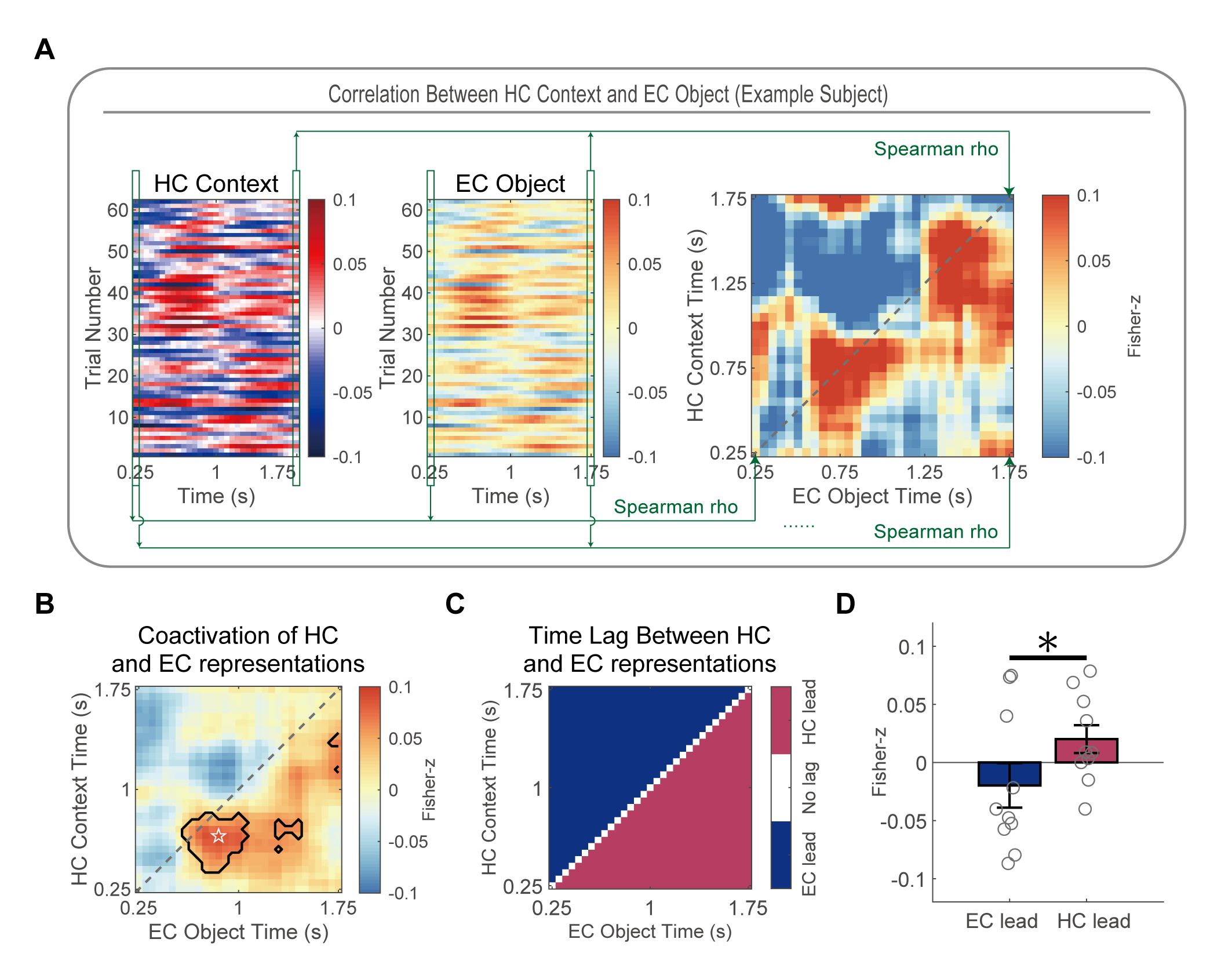

Supplement: S21 Fig — (A) Data from one example subject. Trial-wise time-resolved representations of HC context (the left panel, of dimensions [trials × time windows]) and EC object (the middle panel, of dimensions [trials × time windows]), along with their time × time correlation matrix (the right panel, of dimensions [time windows × time windows]), computed using Spearman rho across trials. Warm colors in the matrix indicate positive correlations between HC and EC representation strength at corresponding time windows. (B) Group-level average of the time × time correlation matrix shown in (A). The black solid lines highlight time window pairs of significant co-activation between HC context and EC object representations. The dashed line denotes the identity line (no time lag). The white star marks the peak correlation. (C) Time-lag map corresponds to (B). Co-activation of HC and EC representations observed in blue regions denotes that EC representations precede HC representations. Conversely, co-activation observed in red regions indicates that HC representations precede EC representations. (D) An across-subject comparison of Fisher z-transformed peak correlation values in EC-leads-HC versus HC-leads-EC regions revealed significantly stronger co-activation when HC led EC. * p < 0.05. (TIF) [file pbio.3003398.s021.tif]

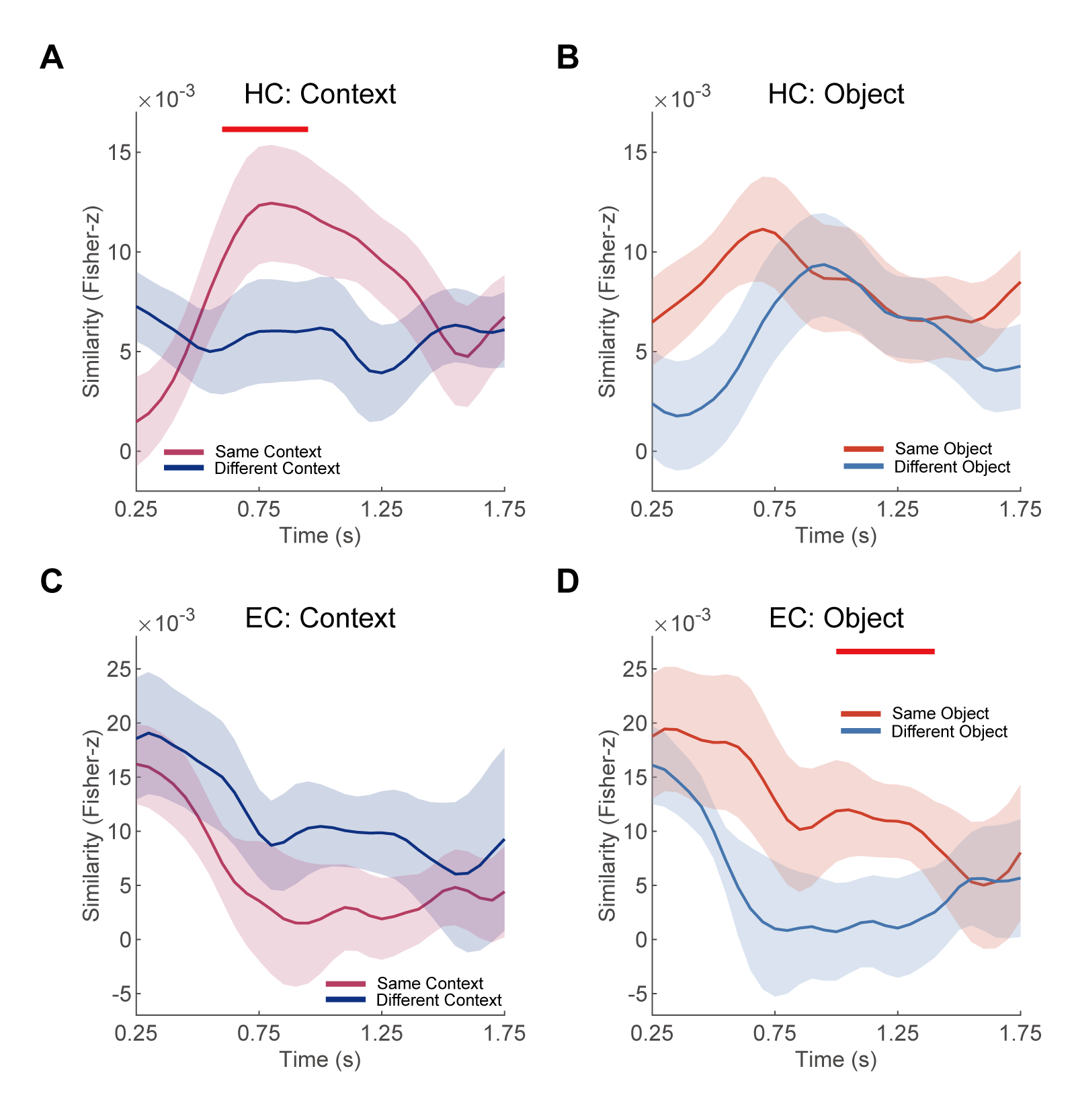

Supplement: S22 Fig — (A) For the translation epochs from good trials, the similarity of Same Context was significantly greater than the similarity of Different Context in HC. The horizontal red line marks the time window where these significant differences were observed. (B) For the translation epochs from good trials, there was no significant difference between the similarity of Same Object and the similarity of Different Object in HC. (C) For the translation epochs from good trials, there was no significant difference between the similarity of Same Context and the similarity of Different Context in EC. (D) For the translation epochs from good trials, the similarity of Same Object was significantly greater than the similarity of Different Object in EC. The horizontal red line indicated the time window where these significant differences were observed. The shaded areas represent SEM across participants. (TIF) [file pbio.3003398.s022.tif]

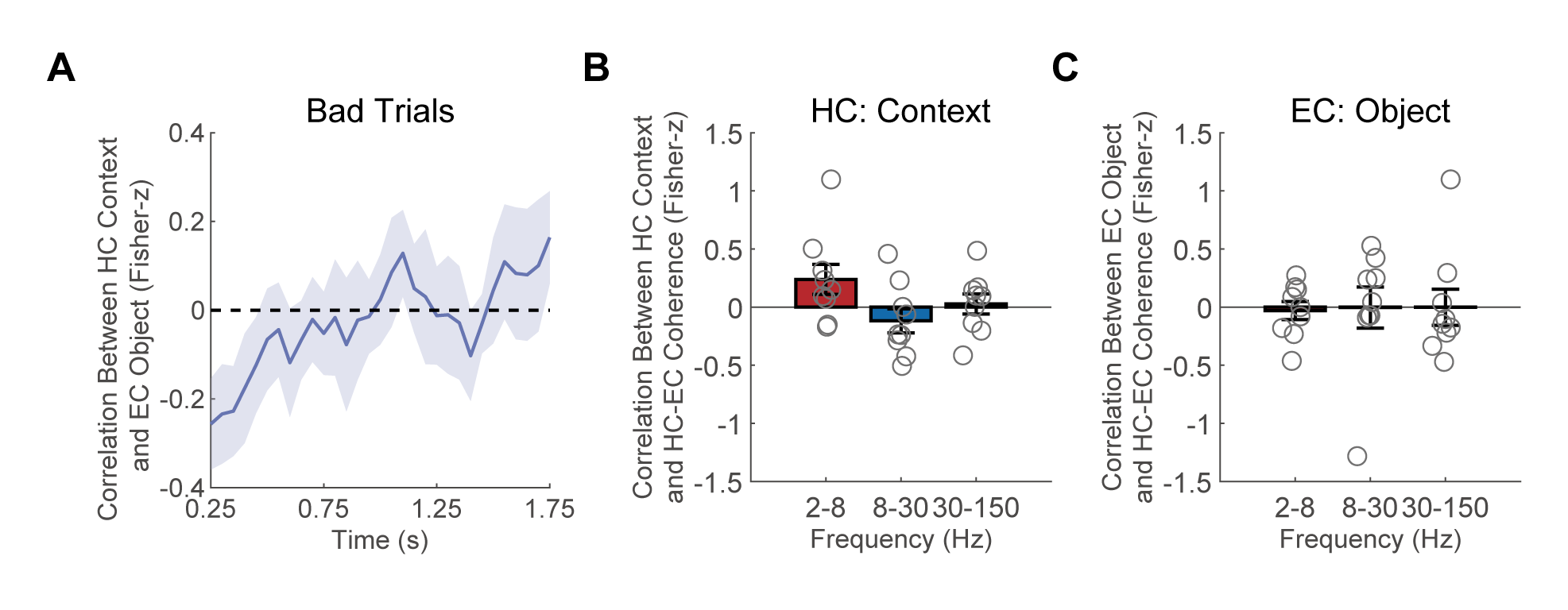

Supplement: S23 Fig — (A) No significant positive correlation was observed between HC context representation and EC object representation in bad trials. (B) In bad trials, HC context representation did not show significant correlations with functional connectivity between HC and EC in any frequency bands. (C) In bad trials, EC object representation did not show significant correlations with functional connectivity between HC and EC in any frequency bands. The shaded areas represent SEM across participants in panel A. The hollow circles indicate individual participant, and error bars indicate SEM across participants in panels B and C. (TIF) [file pbio.3003398.s023.tif]

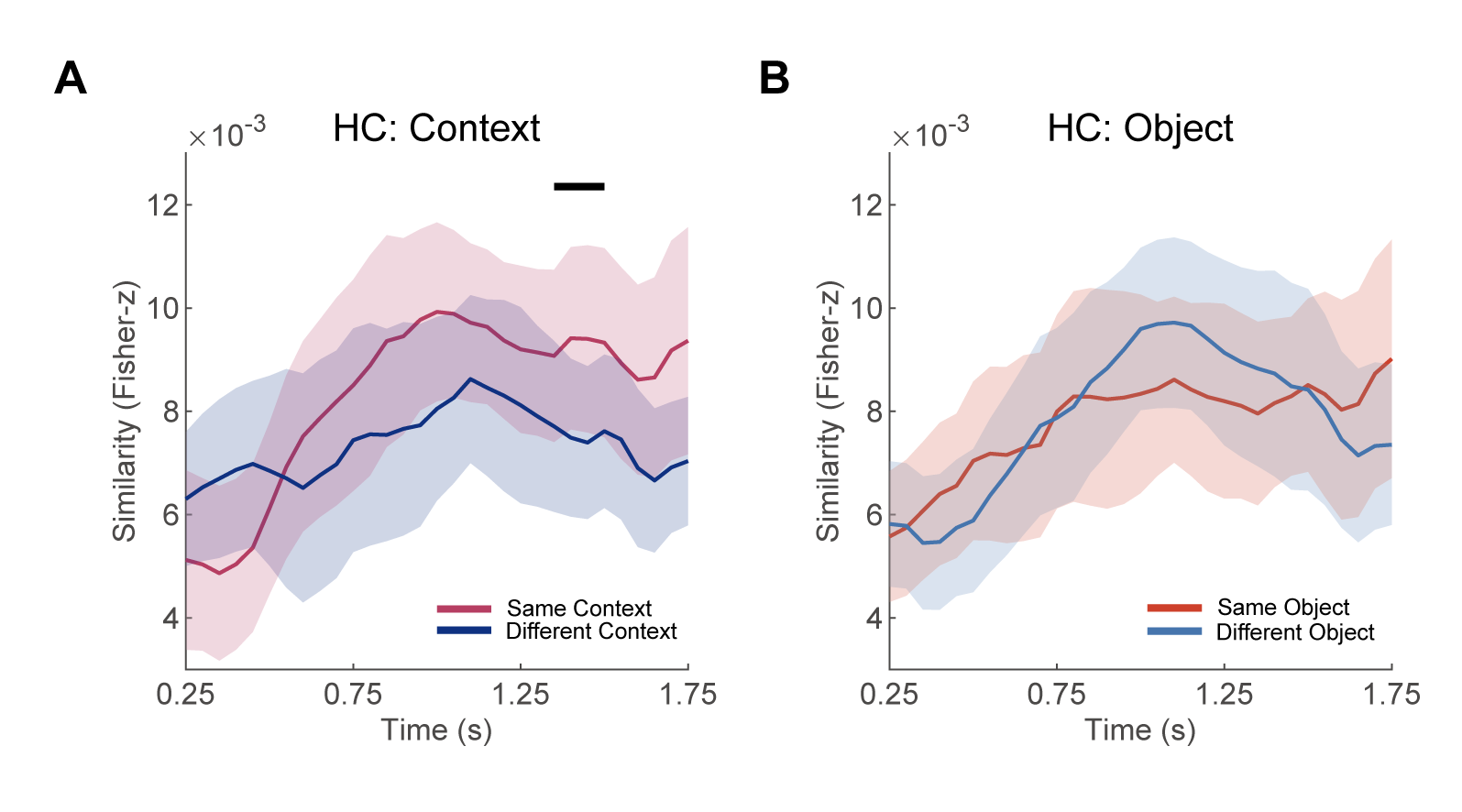

Supplement: S24 Fig — (A) The similarity of Same Context (the red line) was significantly greater compared to the similarity of Different Context (the blue line) in HC. The horizontal black line marked the time window where these significant differences were observed before multiple comparison correction (pcluster = 0.13). (B) During the translation epochs, HC did not exhibit object representation. The shaded areas represent SEM across participants. (TIF) [file pbio.3003398.s024.tif]

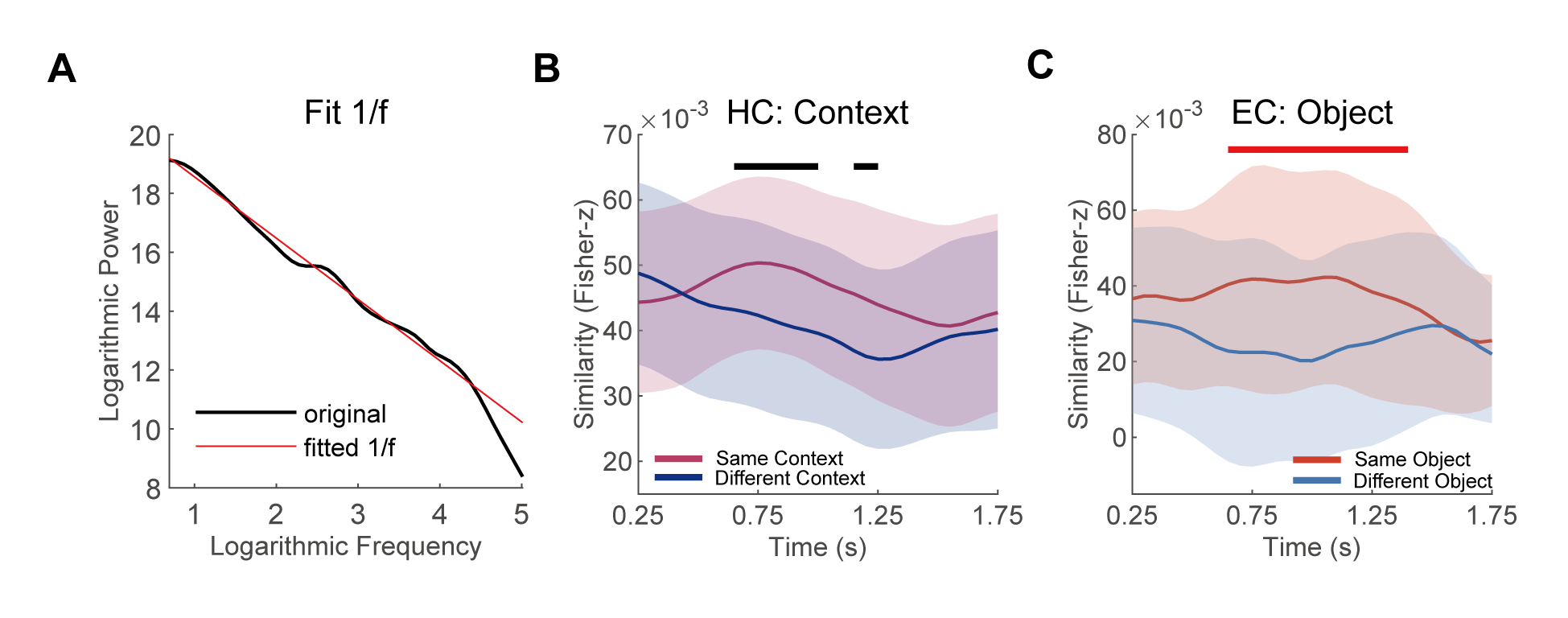

Supplement: S25 Fig — (A) We computed the power spectrum of translation epoch and transformed it into log–log space using the natural logarithm (the black line). A line was then fit to this spectrum in log–log space using a robust regression (the red line). This fitted line was transformed back into linear space to derive the aperiodic component of the signal, which was subsequently subtracted from the original spectrogram to isolate the periodic (oscillatory) power. Finally, we repeated our core RSA using this purified 2–8 Hz oscillatory power. (B) After remove non-oscillatory activity, theta power (2–8 Hz) in HC still represents the context information (cluster 1: 0.65–1 s, pcluster = 0.065; cluster 2: 1.15–1.25 s, pcluster = 0.185). The horizontal black lines mark the time windows showing a marginal significance, as identified by cluster-based permutation test. (C) After remove non-oscillatory activity, theta power (2–8 Hz) in EC still represents the object information (pcluster = 0.001). The horizontal red line marks the time window identified by the cluster-based permutation test. The shaded areas represent SEM across participants in panels B and C. (TIF) [file pbio.3003398.s025.tif]
