# Supplementary material for: Coordinated hippocampal–entorhinal representations support human context-dependent spatial navigation
Source: PLoS Biol. 2025 Sep 17;23(9):e3003398. doi: 10.1371/journal.pbio.3003398 (PMC12453184; doi:10.1371/journal.pbio.3003398)
Supplement: S1 Table — (DOCX) [file pbio.3003398.s026.docx]

**Supplementary Tables**

**S1 Table. Electrode number.**

| ID | HC (All)（）） | HC (S) | EC (All) | EC (S) | AMY (All) | AMY (S) |
| --- | --- | --- | --- | --- | --- | --- |
| Sub01 | 6 | 4 | 5 | 4 | 0 | 0 |
| Sub02 | 5 | 5 | 4 | 4 | 5 | 5 |
| Sub03 | 0 | 0 | 0 | 0 | 0 | 0 |
| Sub04 | 4 | 3 | 0 | 0 | 4 | 2 |
| Sub05 | 3 | 3 | 0 | 0 | 5 | 5 |
| Sub06 | 10 | 10 | 2 | 2 | 6 | 2 |
| Sub07 | 0 | 0 | 0 | 0 | 0 | 0 |
| Sub08 | 8 | 6 | 2 | 2 | 4 | 2 |
| Sub09 | 5 | 4 | 0 | 0 | 4 | 4 |
| Sub10 | 8 | 6 | 0 | 0 | 0 | 0 |
| Sub11 | 0 | 0 | 0 | 0 | 0 | 0 |
| Sub12 | 0 | 0 | 0 | 0 | 0 | 0 |
| Sub13 | 6 | 5 | 0 | 0 | 5 | 5 |
| Sub14 | 7 | 4 | 3 | 3 | 5 | 4 |
| Sub15 | 10 | 5 | 4 | 3 | 0 | 0 |
| Sub16 | 1 | 1 | 0 | 0 | 0 | 0 |
| Sub17 | 9 | 8 | 0 | 0 | 4 | 4 |
| Sub18 | 4 | 4 | 0 | 0 | 0 | 0 |
| Sub19 | 16 | 13 | 0 | 0 | 5 | 4 |
| Sub20 | 7 | 6 | 0 | 0 | 5 | 4 |
| Sub21 | 12 | 12 | 1 | 1 | 4 | 4 |
| Sub22 | 7 | 4 | 1 | 1 | 0 | 0 |
| Sub23 | 0 | 0 | 0 | 0 | 0 | 0 |
| Sub24 | 12 | 11 | 1 | 0 | 8 | 7 |
| Sub25 | 0 | 0 | 0 | 0 | 0 | 0 |
| Sub26 | 8 | 6 | 0 | 0 | 0 | 0 |
| Sub27 | 8 | 7 | 2 | 2 | 5 | 5 |
| Sub28 | 2 | 2 | 0 | 0 | 0 | 0 |
| Sub29 | 14 | 12 | 0 | 0 | 3 | 2 |
| Sub30 | 8 | 8 | 0 | 0 | 5 | 4 |
| Sub31 | 8 | 6 | 3 | 1 | 3 | 3 |
| Total Ele | 188 | 155 | 28 | 23 | 80 | 66 |
| Total Sub | 25 | 25 | 11 | 10 | 17 | 17 |

*Ele: Electrode contacts. Sub: Subjects. S: task-selective electrodes for RSA results reported in main text. Note that 6 patients do not have electrodes in these regions, which results in 25 patients entered for iEEG data analysis.
